# Differences in anxiety sensitivity and experiential avoidance between subtypes of social anxiety disorder

**DOI:** 10.1371/journal.pone.0290756

**Published:** 2023-09-15

**Authors:** Martin Stork, Mariantonia Lemos, Juan Pablo Román-Calderón

**Affiliations:** Department of Psychology, Faculty of Arts and Humanities, EAFIT University, Medellín, Antioquia, Colombia; Medical University of Vienna, AUSTRIA

## Abstract

Both anxiety sensitivity (AS) and experiential avoidance (EA) have been linked to social anxiety disorder (SAD). However, previous studies did not consider their joint variance and the heterogeneity of SAD. In this mixed methods cross-sectional survey, we examined 121 online participants (age range: 16–70 years) who self-reported as socially anxious. We compared AS and EA levels in individuals with a primary fear of noticeable anxiety symptoms vs. behaving ineptly. AS and EA were highly prevalent across the sample. Surprisingly, the noticeable symptoms subtype showed slightly lower AS and EA levels than the inept behavior subtype. The noticeable symptoms subtype scored notably lower on social anxiety measures (mean = 69.8) than the inept behavior subtype (mean = 89.3). EA was uniquely associated with social anxiety in both subtypes, while AS was uniquely associated with social anxiety only in the inept behavior subtype. The joint variance explained substantially more of the noticeable symptoms subtype’s social anxiety (32.5%) compared to the inept behavior subtype’s (9.4%). Qualitative themes aligned with these findings, indicating a self-reinforcing dynamic between high AS, high EA, and social anxiety symptoms. Potential clinical implications are discussed. Future research should examine causality in the AS-EA-SAD dynamic, considering the heterogeneity of SAD.

## Introduction

Social anxiety disorder (SAD) is a mental health diagnosis characterized by excessive fear of being judged, rejected, or negatively evaluated in social or performance situations [1]. With a lifetime prevalence of 4% across all countries, it represents a major clinical and public health burden on a global scale [2]. Although low levels of social anxiety can aid in conveying a positive impression, high social anxiety often creates a paradox. The anxiety reaction, intended to protect individuals from negative social consequences, can inadvertently lead to self-perceived unattractiveness and may cause others to perceive these individuals as less deserving of investing social resources in to form a meaningful relationship with [3]. Most individuals with SAD are aware that their anxious behaviors and symptoms may be viewed unfavorably by others, which easily exacerbates their fear of experiencing anxiety-related symptoms in social situations [4]. In their cognitive model of SAD, Clark and Wells point out how internal events, such as rapid heartbeat, tightness in the throat, or a trembling voice, can be seen as a threat by the person experiencing them, as these symptoms may be noticed by others and lead to negative evaluation [5].

Anxiety sensitivity (AS), defined as the belief that anxiety and its physiological symptoms may have harmful consequences, has been found to be a common phenomenon among socially anxious people [e.g., 6–8]. For example, an individual may be convinced that shaking hands could indicate to others that they are nervous and thus could elicit negative evaluation. An important feature of AS is the early detection of anxiety-related bodily sensations, such as a racing heartbeat, perspiration, or "butterflies" in the stomach [9]. People with elevated levels of AS pick up on these subtle changes more easily and experience them as more uncomfortable than people with low levels of AS.

Moreover, many socially anxious individuals attempt to modify their anxiety and its associated bodily sensations [10]. Attempts to escape, avoid, or alter uncomfortable internal experiences, such as thoughts, emotions, or physical sensations, and to take deliberate actions to decrease their intensity and frequency of occurrence, are encompassed under the umbrella of experiential avoidance (EA) [11, 12]. It has been found that EA readily provokes paradoxical effects, increasing the intensity and occurrence of the internal events a person is trying to prevent [13]. Numerous studies have linked EA with social anxiety, reporting significant positive associations [e.g., 14–17]. These findings have contributed to important adjustments in the treatment of SAD, resulting in an increased implementation of mindfulness- and acceptance-based interventions [e.g., 14]. Recently, Asher, Hofmann, and Aderka identified a reciprocal relationship between social anxiety and EA, with both variables significantly predicting changes in the other [10]. This bidirectional relationship was only observed in individuals with SAD, not in those without. The authors hypothesized that the completion of this harmful EA-social anxiety cycle may represent a crucial distinguishing factor between individuals with and without SAD.

Despite their conceptual fit, only two studies to date have simultaneously examined AS and EA in relation to social anxiety. One study found a significant unique relationship between EA and social anxiety, while the AS-social anxiety relationship was statistically nonsignificant [18]. The other study suggested that EA partially mediates the AS-social anxiety link, indicating that AS predicts social anxiety when individuals engage in EA [19]. However, these studies did not consider the joint predictive properties of AS and EA, nor the heterogeneity of SAD. The exclusive focus on unique prediction often overlooks potentially important processes that lead to correlations [20]. It seems important to examine the shared predictive qualities of AS and EA, rather than just their independent effects, because these variables may interact and have a combined effect on social anxiety that is not captured by examining them separately. Consistent with existing literature [21–23], this study treats AS and EA as distinct but closely related constructs, aiming to explore their combined contributions to social anxiety.

Moreover, it is worth acknowledging that the existing literature has not yet thoroughly explored the nuanced diversity within SAD while investigating AS and EA. Overlooking potential variations among individuals with SAD may lead to misguided assumptions, potentially impacting the effectiveness of treatments and clinical decision-making [24]. While individuals with SAD share a core fear of negative evaluation, rejection, judgment, and disapproval, the intensity and specific focus of these fears can vary among individuals. For instance, while social anxiety typically revolves around concerns of eliciting negative evaluation through one’s behavior, research has pointed to instances where visible signs of anxiety become the primary focus for some individuals [25, 26]. In such cases, affected individuals are primarily preoccupied with their physical arousal and bodily reactions, leading us to hypothesize that this heightened focus on anxiety symptoms may result in elevated levels of AS and EA compared to those with other primary fears. Spokas and Cardociotto have proposed a classification system based on the foci of social fears, delineating subtypes centered on inept behavior, noticeable anxiety symptoms, and causing discomfort to others [24, 27]. While these subtype proposals offer valuable perspectives, empirical data are necessary to firmly establish their validity. Additionally, there currently lacks an empirically supported assessment tool to determine the primary focus of a person’s social fears, aside from directly inquiring with affected individuals. In this context, our study aims to contribute to the argument in favor of intensified research efforts concerning this classification system.

This study aims to fill important knowledge gaps by testing three hypotheses related to AS, EA, and social anxiety. We hypothesize that (1) AS and EA both uniquely as well as jointly predict social anxiety in all subtypes; (2) the noticeable symptoms subtype scores highest on AS and EA measures and these constructs predict social anxiety to a greater extent in this subgroup; and (3) participants’ subjective reports will support these hypotheses, indicating a common pattern of experiential avoidance when facing unwanted anxiety reactions, which is expected to contribute to increased anxiety symptoms for most individuals.

## Materials and methods

### Participants

We conducted a mixed methods self-administered cross-sectional survey questionnaire online, recruiting participants through a multilingual website, ConquerSocialAnxiety.com (https://www.conquersocialanxiety.com/), which offers science-based information on social anxiety. The website is accessible through online search engines, where its articles are listed as search results for specific queries related to the challenges of living with social anxiety. Most website visitors are from Germany, followed by the United States, and represent a broad spectrum of socially anxious individuals from all walks of life. During a 7-week period, from September 1 to October 17, 2022, website visitors were invited to participate in the research study. The relatively short fielding of the survey aimed to collect a sufficient sample size within the available timeframe, considering the study’s project timeline and available resources. Eligible participants were required to self-report being socially anxious and possess sufficient proficiency in English, German, Spanish, Portuguese, or French to complete the survey. The questionnaire was made available in all five languages using validated translations for outcome measures to ensure comparability of results.

### Ethics approval statement

Participants were provided an informed consent form that clearly stated that participation was voluntary, that they could opt out at any time, and that they could choose not to answer any of the questions. They were informed that their responses would be analyzed and reported without the inclusion of data that might reveal their identity. Data were collected using Microsoft Forms, preserving participants’ anonymity. Recognizing the legal provisions in some countries that allow 16 and 17-year-olds to provide informed consent for research participation, we extended the opportunity to participate in the study to this age group in certain regions. As a result, several adolescents were included in the research cohort. Given that the present study included questions that could provoke emotional responses, it was submitted to the Ethics Committee of EAFIT University, Medellín, Colombia. It was classified as a low-risk study and received the approval of said committee.

### Instruments and procedure

The survey included three primary outcome measures. First, we included the Liebowitz Social Anxiety Scale-Self-Report version (LSAS-SR) and its respective validated translations to capture respondents’ level of fear and anxiety in 24 social situations, along with their tendency to avoid these situations through a 4-point agreement/frequency Likert scale [28]. The LSAS-SR has been shown to be a reliable instrument for assessing social anxiety in both clinical and nonclinical samples [29]. The LSAS-SR has high internal consistency with a Cronbach’s α-value of 0.95, strong twelve-day test-retest reliability (r = 0.83), and exhibits robust convergent and discriminative validity [30]. In the present study, the internal consistency of the LSAS-SR was strong (Cronbach’s α = 0.96).

Second, we used the second version of the Acceptance and Action Questionnaire (AAQ-II) and its respective validated translations to measure participants’ degree of EA [31, 32]. The AAQ-II consists of 7 questions that are rated on a 7-point agreement Likert scale, and assesses psychological inflexibility, which allows for determination of a person’s EA. It has shown good internal consistency with a Cronbach’s α value of 0.88, good test-retest reliability at 3 months (0.81) and 12 months (0.79), and adequate convergent and discriminative validity [31]. The internal consistency of the AAQ-II in the present sample was high (Cronbach’s α = 0.90).

Third, we included the Anxiety Sensitivity Index-3 (ASI-3) and its respective validated translations to assess participants’ apprehension about anxiety symptoms [33]. It consists of 18 items that are scored on a 5-point magnitude Likert scale, assessing physical, cognitive, and social concerns, allowing to determine a person’s sensitivity to anxiety-related symptoms. It has shown excellent reliability for the total score (Cronbach’s α = 0.93) and good internal consistency for each of its three lower-order factors, with α values ranging from 0.76 to 0.86 for the physical concerns subscale, from 0.79 to 0.91 for the cognitive concerns subscale, and from 0.73 to 0.90 for the social concerns subscale [34]. The ASI-3 also presents good convergent and discriminative validity. The overall internal consistency of the ASI-3 in the present sample was strong (Cronbach’s α = 0.92).

As a secondary outcome measure, we included a qualitative component with open-ended questions specifically designed for this study. Participants were invited to provide detailed accounts of their subjective experiences with intrusive internal events that occur during social situations. These events encompassed unwanted or uncomfortable thoughts, feelings, and bodily reactions or physical manifestations of anxiety. Additionally, participants were asked to describe how they typically responded to these unwanted experiences during social situations. Through these inquiries, we aimed to gain insights into the impact of their responses on their anxiety or nervousness in social settings. To access the full questionnaire, including the open-ended questions used in this study, as well as the quantitative and qualitative data collected and the output data from our analyses, please refer to the repository available at https://repository.eafit.edu.co/handle/10784/32174.

Lastly, participants were directly queried regarding the specific focus of their social fears, enabling us to categorize them into distinct subtypes, namely, those exhibiting a primary fear of behaving ineptly, showing noticeable anxiety symptoms, offending others, or participants who did not fall into any of these subtype categories. It is important to highlight that currently, no standardized and empirically supported screening tool exists for social anxiety subtypes based on the focus of social fears. As a result, we employed direct inquiries, adopting a qualitative approach for subtype classification.

The reporting of this cross-sectional survey study adheres to the STROBE checklist, which provides guidelines for the transparent reporting of observational studies in epidemiology.

### Power analysis

In conducting the power analysis, we encountered challenges in finding studies that directly measured the relationship between AS and EA with social anxiety using the LSAS-SR. Although we found two relevant studies, one measured social anxiety using a different instrument, the Social Interaction Phobia Scale (SIPS), and the other used the Beck Anxiety Inventory (BAI), which is not specific to social anxiety. To err on the conservative side, we adopted the lowest effect sizes reported in these studies for AS and EA in our power analysis. This approach was chosen to ensure a cautious estimation of effect sizes given the limited availability of exact measures.

To determine the appropriate sample size for the study and ensure sufficient statistical power, power analyses for both predictors, EA and AS, were conducted using G*Power v. 3.1 software [35, 36]. For the AS predictor, we used an effect size (f2) of 0.214168, an alpha error probability of 0.025 (with Bonferroni correction for two hypotheses), a total sample size of 121, and a model with two tested predictors out of a total of four predictors. The output from the power analysis for the AS predictor indicated a noncentrality parameter (A) of 25.9143280, a critical F value of 3.8087154, numerator degrees of freedom (df) of 2, denominator df of 116, and a high statistical power (1-ß err prob) of 0.9924174. Similarly, for the EA predictor, we used an effect size (f2) of 0.1976936, an alpha error probability of 0.025 (with Bonferroni correction for two hypotheses), a total sample size of 121, and a model with two tested predictors out of a total of four predictors. The output from the power analysis for the AS predictor revealed a noncentrality parameter (A) of 23.9209256, a critical F value of 3.8087154, numerator degrees of freedom (df) of 2, denominator df of 116, and a high statistical power (1-ß err prob) of 0.9872921. These power analyses confirm that the study is adequately powered to detect the effects of interest for both AS and EA predictors.

### Rationale for research design

We adopted a mixed methods approach to capitalize on the strengths of both quantitative and qualitative data, allowing for a comprehensive understanding of the interplay between social anxiety, AS, and EA in diverse social anxiety subtypes. While quantitative data provided valuable insights, the inclusion of a qualitative component allowed participants to directly express their experiences, enriching our understanding of how AS and EA influence social anxiety and vice versa. This facilitated an in-depth exploration of participants’ lived experiences through deductive thematic analysis, providing insights into the interplay between social anxiety and heightened AS and EA.

### Analysis

We conducted a correlation analysis to examine the relationship between EA, AS, and social anxiety, using social anxiety as the dependent variable. Next, we performed multiple linear regression analyses, also using social anxiety as the dependent variable, to investigate the relationship between EA, AS, and social anxiety. We analyzed the complete sample and additionally the inept behavior and noticeable symptoms subtypes as separate groups. Due to small sample sizes, we were unable to analyze the offensive subtype (N = 6) and participants who did not identify with any of the proposed foci of social fears (N = 4). However, these groups were included in the analysis of the complete sample.

The regression included the three subscales of AS separate independent variables to examine whether the social concerns subscale played a more significant role than the physical and cognitive subscales. To determine the relative strength of EA, AS, and each of the AS subscales as unique and joint predictors of social anxiety, we computed the squared semipartial correlations. To control for potential confounding effects on EA and AS, we introduced participants’ age and income classification of their countries as control variables. Age has been found to be inversely correlated with social anxiety [37, 38], while social anxiety is more prevalent in high-income countries [2]. This analysis was performed with IBM SPSS Statistics for Windows, Version 29.0 (RRID:SCR_002865).Next, we thematically analyzed the qualitative data using an inductive method. We assigned eligible responses to one of three categories: (1) intrusive internal events, (2) reactions to these intrusive events, and (3) the effects of these reactions. Data were analyzed in their original languages. For communication purposes, direct quotations presented in the qualitative results section that were provided in German, Spanish, Portuguese, or French were translated into English. This part of the analysis was performed with ATLAS.ti for Windows, Version 8.0 (RRID:SCR_022920).

## Results

### Description of the sample

Initially, we received 125 valid responses from participants. However, we excluded 4 participants (3.2%) due to a lack of data, resulting in a final sample size of 121 individuals. This final sample comprised 113 adults (93.39%) and 8 adolescents (6.61%). The mean age was 30.8 years (S.D. = 12.0) and the age span ranged from 16 to 70 years old. The most common nationalities were German (49.59%), US American (13.22%), Colombian (4.96%), and Brazilian (4.96%). More than half of the participants responded in German (52.07%), more than a quarter replied in English (29.75%), and significantly fewer in Spanish (11.57%), with Portuguese (4.13%) and French (2.48%) being the languages with the lowest response rates. 20 respondents (16.53%) lived in middle- or low-income countries according to the World Bank’s classification system [39]. 96 participants (79.34%) lived in high-income countries, and 5 individuals (4.13%) refrained from sharing their country of residence.

### Quantitative results

#### Descriptive statistics

The mean social anxiety score in the present sample was 81.0 (S.D. = 28.1), with most subjects (75.2%) reaching or exceeding the suggested cut-score of 60, at which the presence of SAD is likely [29] (Table 1). About a quarter of the participants (24.8%) achieved lower scores. As classified by the LSAS-SR, 44 participants (36.4%) had very severe social anxiety, 22 participants (18.2%) had severe social anxiety, 18 participants (14.9%) had marked social anxiety, 17 participants (14.1%) had moderate social anxiety, 14 participants (11.6%) had mild social anxiety, and 6 participants (5.0%) had no social anxiety. The mean social anxiety score for the inept behavior subtype was 89.3 (S.D. = 23.1), while for the noticeable symptoms subtype, it was 69.8 (S.D. = 31.8).

**Table 1:** Descriptive statistics of social anxiety, AS, and EA.

	Mean (S.D.)
Social Anxiety	
Complete Sample	81.0 (28.1)
Inept Behavior Subtype	89.3 (23.1)
Noticeable Symptoms Subtype	69.8 (31.8)
Experiential Avoidance	
Complete Sample	33.9 (9.6)
Inept Behavior Subtype	36.3 (8.1)
Noticeable Symptoms Subtype	30.9 (11.0)
Anxiety Sensitivity	
Complete Sample	37.5 (15.9)
Physical Concerns	9.8 (6.4)
Social Concerns	17.4 (5.2)
Cognitive Concerns	10.3 (6.9)
Inept Behavior Subtype	40.6 (16.7)
Physical Concerns	10.5 (6.8)
Social Concerns	18.2 (5.4)
Cognitive Concerns	11.9 (6.6)
Noticeable Symptoms Subtype	34.5 (14.1)
Physical Concerns	9.1 (5.8)
Social Concerns	17.6 (4.2)
Cognitive Concerns	7.9 (6.8)

S.D. = Standard Deviation

The mean EA score for the entire sample was 33.9 (S.D. = 9.6), with most respondents (86.0%) scoring 24 or higher, a score that the questionnaire authors associate with cut-off scores for clinical symptom measures. The remaining participants (14.1%) scored below. The mean EA score for the inept behavior subtype was 36.3 (S.D. = 8.1), and for the noticeable symptoms subtype, it was 30.9 (S.D. = 11.0).

The mean of the total AS score for the entire sample was 37.5 (S.D. = 15.9). 14 subjects (11.6%) fell into the normative AS category and 9 participants (7.4%) were assigned to the moderate-to-high AS category which was recently proposed [40]. The remaining 98 subjects (81.0%) met or exceeded the cut-off score of 23 to be classified in the high AS category. The means of the three subscales of the ASI-3 were 9.8 (S.D. = 6.4) for physical concerns, 17.4 (S.D. = 5.2) for social concerns, and 10.3 (S.D. = 6.9) for cognitive concerns. For the inept behavior subtype, the mean AS score was 40.6 (S.D. = 16.7), and 10.5 (S.D. = 6.8) for the physical subscale, 18.2 (S.D. = 5.4) for the social subscale, and 11.9 (S.D. = 6.6) for the cognitive subscale. For the noticeable symptoms subtype, the mean AS score was 34.5 (S.D. = 14.1), and 9.1 (S.D. = 5.8) for the physical subscale, 17.6 (S.D. = 4.2) for the social subscale, and 7.9 (S.D. = 6.8) for the cognitive subscale.

A large proportion of participants reported the fear of behaving ineptly as their main concern in social situations (54.6%), followed by those focusing primarily on noticeable signs of anxiety (37.1%). Considerably fewer participants mentioned fear of offending others as their main concern (5.0%) or said their fear did not focus on any of these options (3.3%).

#### Correlation matrix

Prior to conducting multiple linear regression analysis, the relationship between EA, AS, and social anxiety was examined using a correlation matrix (Table 2). The complete socially anxious sample demonstrated significant positive correlations between social anxiety scores and both EA scores (r = 0.601, p < .001) and AS scores (r = 0.509, p < .001). Similar patterns were observed within the inept behavior subtype, where social anxiety scores were significantly positively correlated with EA scores (r = 0.424, p < .001) and AS scores (r = 0.439, p < .001). Furthermore, for the noticeable symptoms subtype, social anxiety scores were also significantly correlated with EA scores (r = 0.710, p < .001) and AS scores (r = 0.551, p < .001).

**Table 2:** Correlation matrix of social anxiety, EA and AS variables.

Group	Social Anxiety	EA	AS
**Complete Sample**			
EA	0.601^a^	-	-
AS	0.509^a^	0.560^a^	-
**Noticeable Symptoms**			
EA	0.710^a^	-	-
AS	0.551^a^	0.622^a^	-
**Inept Behavior**			
EA	0.424^a^	-	-
AS	0.439^a^	0.490^a^	-

EA = Experiential Avoidance; AS = Anxiety Sensitivity.

^a^ p < .001

#### Multiple linear regression analysis

Next, we proceeded with multiple linear regression analyses to investigate the relationship between EA and AS with social anxiety, while controlling for age and country income classification. For the entire socially anxious sample, both EA (β = 1.353, p < .001) and the AS social concerns subscale (β = 1.542, p = .002) were statistically significant predictors of social anxiety, whereas the physical and cognitive concerns factors failed to predict social anxiety, F (6, 109) = 13.339, p < .001, R2 adj = 42.3 (Table 3). Similarly, when analyzing the inept behavior subtype, we found that EA (β = 1.050, p = .004) and the AS social concerns subscale (β = 2.622, p < .001) were statistically significant predictors, whereas the physical and cognitive concerns subscales indicated a lack of statistical significance, F (6, 57) = 6.422, p < .001, R2 adj = 40.3. The joint variance explained by AS and EA accounted for 9.4% of the total variance in social anxiety for this subtype. Finally, for the noticeable symptoms subtype, only EA (β = 1.605, p = .002) proved to be a statistically significant predictor, whereas all lower-order factors of AS, including the social concerns subscale, proved to be statistically non-significant, F (6, 35) = 6.331, p < .001, R2 adj = 52.0. The joint variance explained by AS and EA was relatively higher, at 32.5% for the inept behavior subtype.

**Table 3:** Multiple linear regression analysis for social anxiety, Experiential Avoidance and Anxiety Sensitivity.

Dependent Variable: Social Anxiety (LSAS-SR Score)							
	Independent Variables	B	Standard Error	Beta	T	^a^Sig	^b^R^2^
**Complete Sample**							
Model 1	(Constant)	86.324	8.749	-	9.866	.000	0.4%
	Age	-.097	.218	-.042	-.443	.659	-
	High Income	-2.949	6.873	-.040	-.429	.669	-
Model 2	(Constant)	8.176	11.684	-	.700	.486	-
	Age	.030	.171	.013	.178	.859	-
	High Income	-2.030	5.347	-.028	-.380	.705	-
	EA	1.353	.267	.463	5.067	.000	-
	AS Physical	.245	.479	.057	.512	.609	-
	AS Social	1.542	.492	.291	3.134	.002	-
	AS Cognitive	-.144	.481	-.036	-.300	.765	-
	Total Variance Explained	-	-	-	-	-	42.3%
**Noticeable Symptoms Subtype**							
Model 1	(Constant)	75.591	19.443	-	3.888	.000	0.4%
	Age	.023	.390	.009	.059	.953	0.0%
	High Income	-6.979	16.783	-.067	-.416	.680	0.4%
Model 2	(Constant)	-3.440	22.937	-	-.150	.882	-
	Age	-.057	.299	-.023	-.191	.849	0.0%
	High Income	-3.414	12.470	-.033	-.274	.786	0.1%
	EA	1.605	.466	.550	3.445	.002	16.2%
	AS Physical	.644	.935	.123	.688	.496	0.7%
	AS Social	1.362	1.011	.190	1.347	.187	2.5%
	AS Cognitive	-.095	.926	-.020	-.102	.919	0.0%
	Total Variance Explained	-	-	-	-	-	52.0%
	**Joint Variance Explained**	-	-	-	-	-	**32.5%**
**Inept Behavior Subtype**							
Model 1	(Constant)	89.913	10.206	-	8.810	.000	0.1%
	Age	-.071	.290	-.032	-.245	.807	-
	High Income	1.266	7.194	.023	.176	.861	-
Model 2	(Constant)	12.987	15.917	-	.816	.418	-
	Age	.109	.236	.049	.464	.645	0.2%
	High Income	-1.712	5.806	-.031	-.295	.769	0.1%
	EA	1.050	.353	.354	2.973	.004	9.2%
	AS Physical	.013	.554	.004	.024	.981	0.0%
	AS Social	2.622	.626	.602	4.186	.000	18.3%
	AS Cognitive	-1.010	.583	-.283	-1.733	.088	3.1%
	Total Variance Explained	-	-	-	-	-	40.3%
	**Joint Variance Explained**	-	-	-	-	-	**9.4%**

EA = Experiential Avoidance; AS = Anxiety Sensitivity.

^a^Significant at 0.05

^b^R2 = Explained Variance

The inclusion of age and country income classification as control variables did not significantly alter the relationship between EA, AS, and social anxiety, both in the complete sample and the two subtypes.

### Qualitative results

Finally, we thematically analyzed the qualitative part of the questionnaire, which aimed to explore participants’ subjective experiences regarding the dynamics of AS, EA, and social anxiety symptoms. Participants’ responses were categorized into several themes related to (1) intrusive internal events, such as uncomfortable thoughts, feelings, and physiological reactions, (2) participants’ typical reactions to these events, such as acceptance, distraction, or actively trying to suppress or alter these phenomena, and (3) the effects these reactions tend to have on respondents’ social anxiety symptoms, such as changes in momentary anxiety, physiological arousal, or self-perceptions during social situations.

#### Intrusive internal events

**Fig 1:**
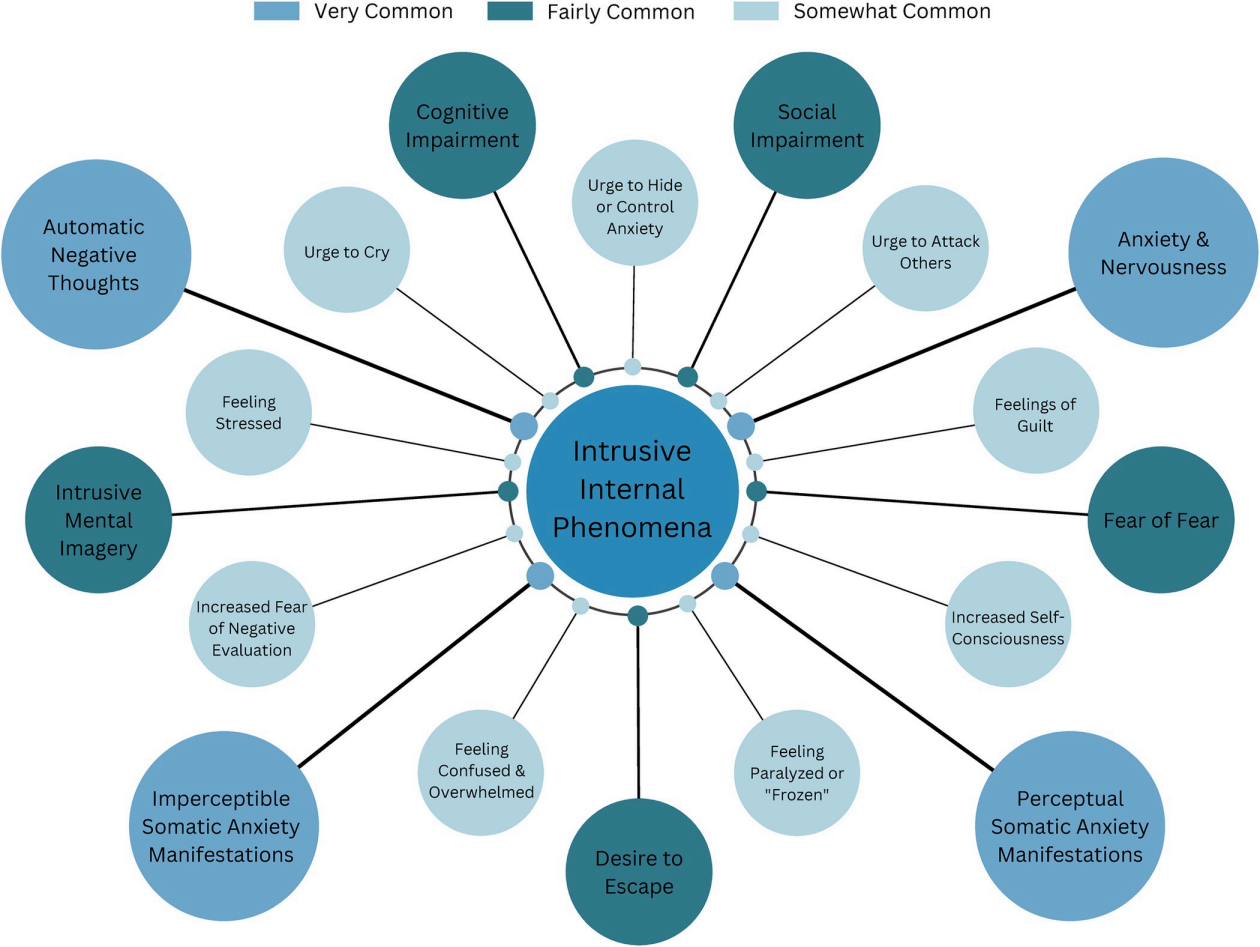
Intrusive internal phenomena in social situations. Note: Bigger Circle Size Indicates Greater Frequency.

Participants described various intrusive internal phenomena experienced during social situations (Fig 1). Common themes included somatic anxiety manifestations, both noticeable and not noticeable to others, feelings of anxiety and nervousness, and automatic negative thoughts. Participants often reported experiencing a range of physical symptoms, such as rapid heartbeat, difficulty breathing, trembling, and dizziness. These experiences were accompanied by a sense of helplessness, confusion, and feeling overwhelmed. Additionally, many participants expressed heightened self-consciousness, feeling as though they were being observed from outside their bodies. Negative thoughts about self-image and concerns about others’ evaluations were also prevalent.

#### Reactions to intrusive internal events

**Fig 2:**
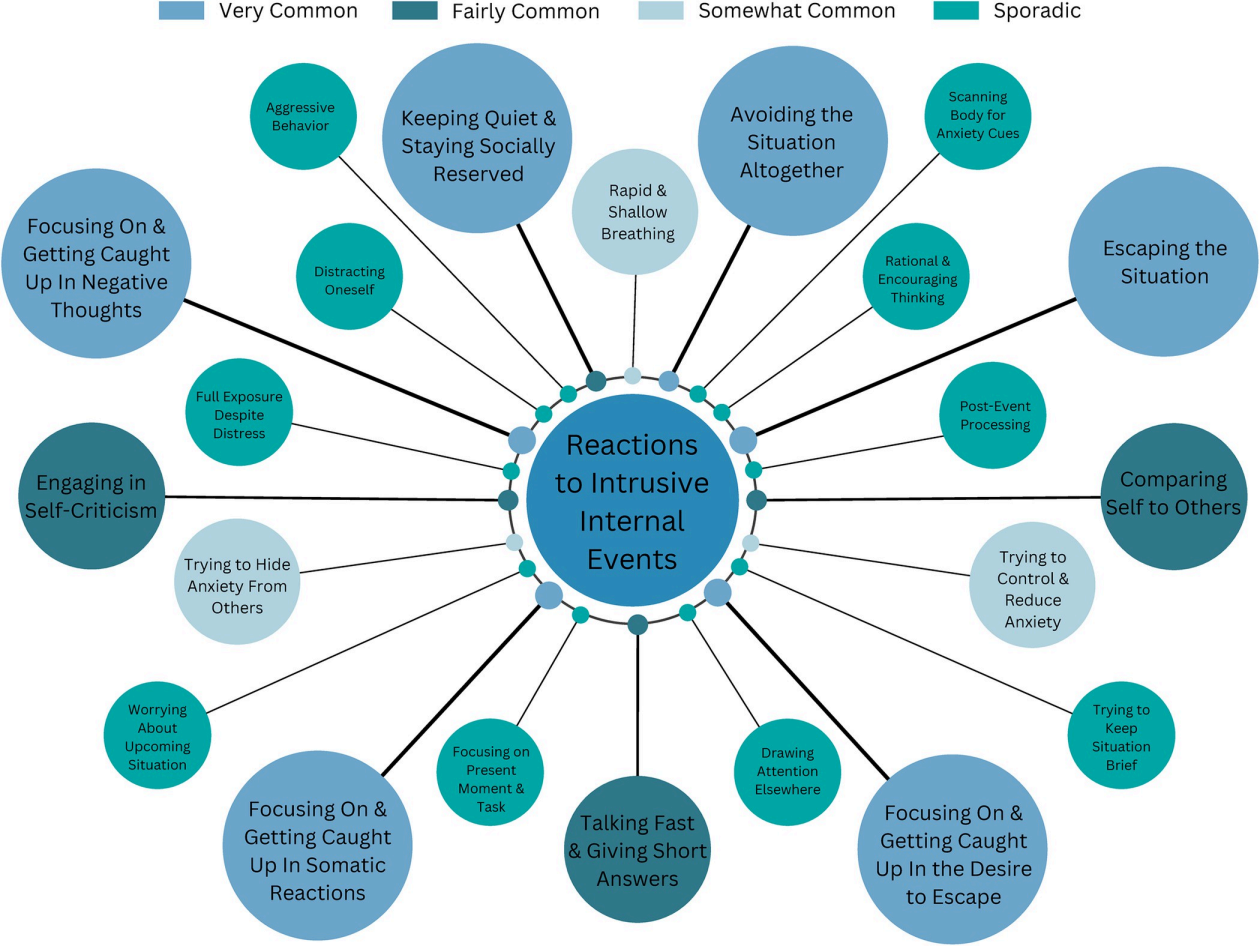
Reactions to intrusive internal phenomena in social situations. Note: Bigger Circle Size Indicates Greater Frequency.

In response to such intrusive internal events, participants reported a tendency to focus on these experiences and get carried away by them (Fig 2). They described automatic acceptance of their negative thoughts as well as assumptions of their truthfulness. Many participants engaged in avoidance as a primary coping mechanism, either by escaping the situation or resorting to safety behaviors, such as hiding physical reactions or speaking only briefly. We also observed strong patterns of anticipatory anxiety and post-event processing, where individuals excessively worried about future situations or ruminated about past social interactions. Respiratory distress and shallow breathing were commonly reported, particularly among those who experienced panic-like symptoms.

#### Effects of reactions to intrusive internal events

**Fig 3:**
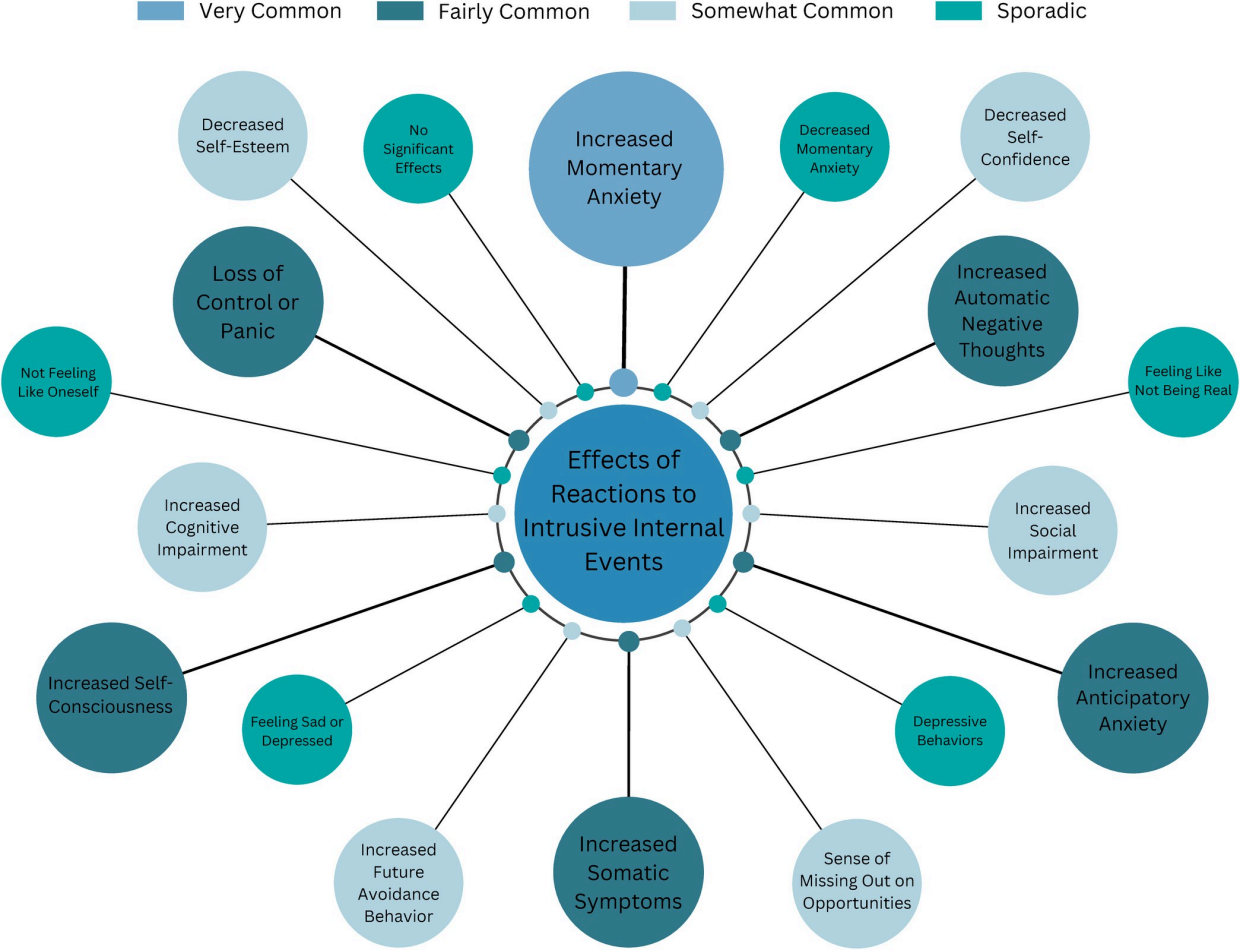
Effects of reactions to intrusive internal phenomena in social situations. Note: Bigger Circle Size Indicates Greater Frequency.

Participants’ reactions to intrusive internal events often resulted in increased momentary social anxiety (Fig 3). Many participants reported that attempts to control, suppress, or conceal anxiety symptoms frequently led to a loss of control and, in some cases, panic attacks. Participants reported an escalation of physical anxiety symptoms, negative thoughts, and self-consciousness as a consequence of their reactions. Increased anticipatory anxiety was also observed, with individuals experiencing heightened fear and worry about upcoming social situations. Long-term effects included decreased self-confidence and self-esteem, increased avoidance behavior, and a sense of missing important opportunities in education and career. Some participants also expressed feelings of not being authentic and reported experiencing depressive symptoms.

#### AS-EA-social anxiety interplay

Participants’ experiences with intrusive internal events during social situations unveiled a profound struggle between the fear of experiencing anxiety symptoms (AS), attempts to prevent, reduce, or alter any emerging anxiety symptoms (EA), and the subsequent exacerbation of social anxiety symptoms themselves. The following accounts by participants exemplify this dynamic.

“*I usually try to calm myself down and mend the situation*, *but almost always end up making things even worse and stress myself out more*, *leading to a full panic attack with hyperventilation and shaky voice*.*”*—Greek participant, young adult

"*I often fall into a vicious cycle in which my physical symptoms feed my worries*, *which in turn feed my physical symptoms*, *and this cycle escalates to the point where I need to get out of the situation*.*"*—Brazilian participant, young adult

“*At university*, *in seminars*, *or in rounds of introductions*, *it often happens that we sit in a circle and everyone has to say something in turn*. *I can’t escape from this situation and the tension increases more and more until it’s my turn*. *My heart starts to beat louder and louder and I have a roaring in my ears*, *I cannot hear anything else at this point*. *It is often difficult for me to breathe and when time comes for me to say something*, *my voice often trembles*, *or I don’t have enough air to speak*.*”–*German participant, young adult

“*[…]*. *This stems from an incident many years ago when I had a panic attack [*…*]*. *I panicked and lost even more control of my legs*. *Then*, *as I struggled to walk*, *two girls caught my attention*, *and they were laughing at me*. *I was shocked*, *embarrassed*, *and humiliated*. *From then on*, *I started to look down at myself*, *checking myself*, *and whenever I felt the slightest feeling in my legs*, *I panicked thinking that people will notice and ridicule me*. *This has happened for the last 30 years*.*”–*British participant, middle-aged adult

“*I try to control myself*, *but my biggest fear is not being able to*. *For example*, *just yesterday in a meeting I was asked to read a text and*, *on the 4th or 5th line my breathing became so agitated that I said my vision was blurred and asked someone to read it for me*.”–Brazilian participant, middle-aged adult.

### Joint analysis

**Fig 4:**
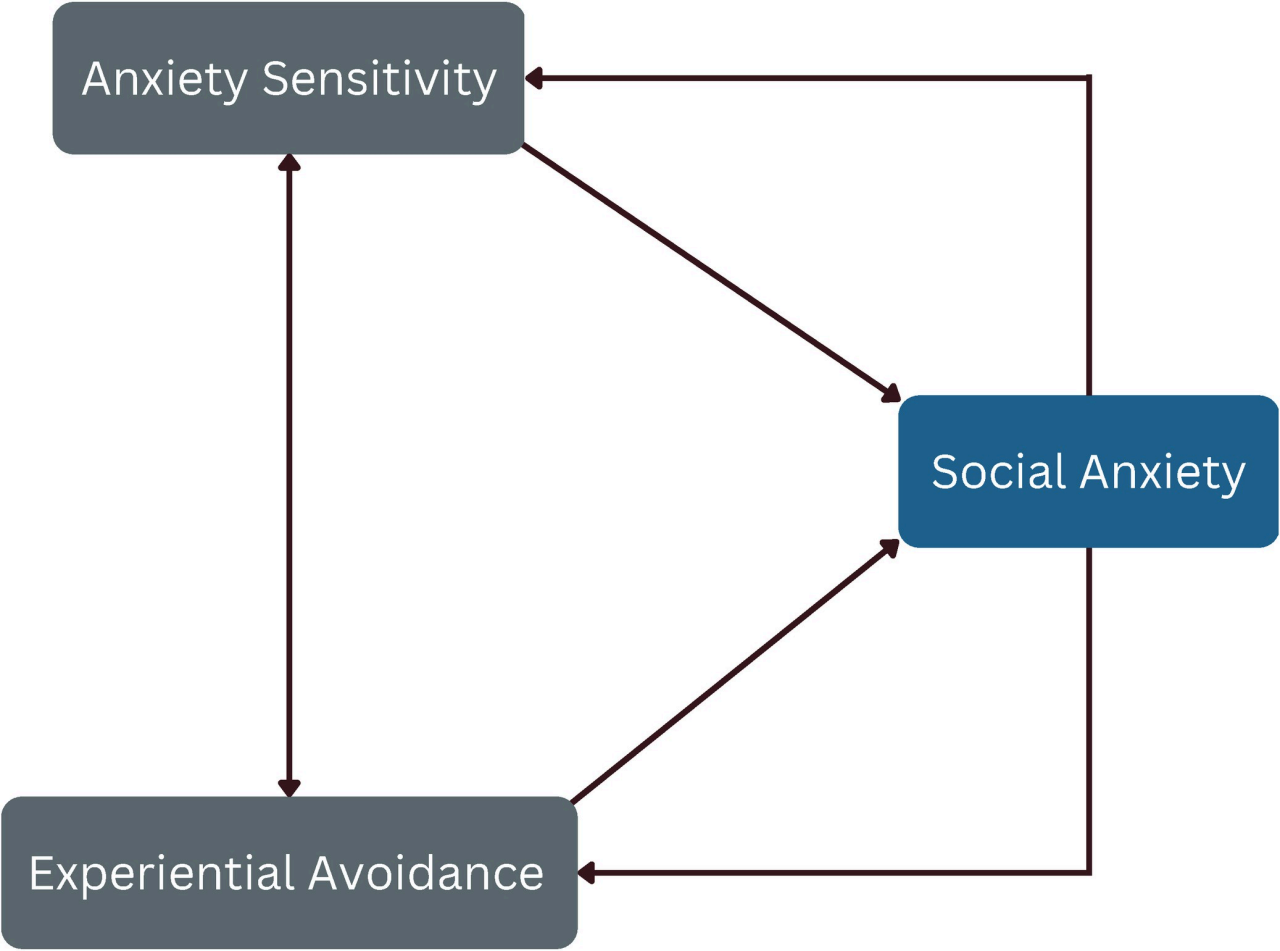
Proposed self-reinforcing development system of Anxiety Sensitivity, Experiential Avoidance, and social anxiety.

Our quantitative data indicate that a substantial portion of the explained variance in this two-predictor model is predicted by a joint contribution from AS and EA, with predictive properties varying across subtypes. Moreover, our qualitative analysis shed light on participants’ experiences, indicating that heightened state social anxiety could potentially lead to increased momentary AS and EA. This suggests a dynamic interplay between these constructs, where the experience of social anxiety might trigger elevated levels of AS and EA, and in turn, AS and EA may contribute to intensified social anxiety symptoms. We hypothesize that there may be a self-reinforcing system between AS, EA, and social anxiety, mirroring the development system of two-predictor models proposed by Schoen, DeSimone, and James [20]. This proposed dynamic is visually depicted in Fig 4.

## Discussion

This study aimed to examine the associations of AS and EA with social anxiety symptoms. Our primary interest was in understanding both their individual contributions and their joint ability to predict social anxiety. Additionally, we investigated potential differences between subtypes of social anxiety, hypothesizing that individuals primarily concerned about displaying noticeable anxiety symptoms would not only exhibit stronger associations with AS and EA but also that these constructs would play a more crucial role in predicting social anxiety in this particular subgroup.

While SAD is commonly studied and treated as a homogeneous clinical condition, our exploratory study revealed significant variability among affected individuals. Participants were asked to identify with one of the three proposed subtypes of social anxiety based on the focus of their social fears: inept behavior, noticeable symptoms, or causing discomfort to others. Our findings provide preliminary evidence of important differences between the two studied subgroups, particularly in terms of overall scores on social anxiety measures. The noticeable symptoms subtype scored significantly lower than the inept behavior subtype on these measures, signaling a difference of “marked social anxiety” compared to “severe social anxiety” according to the classification system of the LSAS-SR [28]. Additionally, the combination of AS and EA accounted for a more substantial portion of the social anxiety scores among the noticeable symptoms subtype.

Our study’s findings align with previous research that examined the relationship between AS, EA, and social anxiety in SAD. Consistent with prior studies [18, 19], we observed that for the noticeable symptoms subtype, the link between EA and social anxiety was statistically significant, while the association with AS was not. Conversely, for the inept behavior subtype, both EA and AS emerged as unique predictors of social anxiety variance, indicating a noteworthy divergence between these subtypes.

It is worth noting that one of the previous studies [19] suggested that AS predicts social anxiety only when individuals engage in EA. Our findings complement this idea, as we discovered that the combined effects of AS and EA accounted for a larger proportion of the variance in social anxiety compared to the unique predictive contribution of AS alone. Specifically, for the inept behavior subtype, AS significantly predicted social anxiety independently of EA, whereas for the noticeable symptoms subtype, AS was relevant primarily in conjunction with EA. The joint effects of AS and EA on social anxiety demonstrated a significantly greater variance accounted for in the noticeable symptoms subtype, almost three times as much as the variance in the inept behavior subtype. This discrepancy points to a potentially greater importance of AS and EA for the noticeable symptoms subtype and provides support for the concept of EA potentially mediating the impact of AS [19].

The overall effect sizes for AS and EA were substantial, particularly for the noticeable symptoms subtype, underscoring their significance in understanding social anxiety. Moreover, when considering the significant differences in social anxiety measures between the two subgroups and the distinct role of AS a unique predictor for the inept behavior subtype, our findings advocate for distinguishing between the noticeable symptoms and inept behavior subtypes of SAD.

The observed differences between subtypes may be explained by several factors. For one, the focus of an individual’s social fears is likely to be influenced by their unique negative experiences in social contexts. Individuals who are primarily concerned about behaving inappropriately or being perceived as socially unskilled are likely to have had such negative experiences in the past, leading to this concern. As such, it seems plausible that the inept behavior subtype contains a higher proportion of individuals with deficits in social skills and behavioral inhibition, two constructs related to decreased quality of social interactions that have been consistently linked to social anxiety [41, 42]. This would justify higher measures of social anxiety among this group, since their difficulties would likely permeate more areas of the social domain. On the other hand, individuals who are primarily concerned about showing noticeable signs of anxiety are likely to have had negative experiences in which they were judged for their perceptible anxiety symptoms, thus leading to such a fear [43]. Since the fear of showing noticeable signs of anxiety can be quite circumscribed, for example in public speaking scenarios, it seems plausible that this group of individuals would exhibit more areas of normal social functioning, as compared to the inept behavior subgroup, justifying lower overall scores on social anxiety measures. Second, while AS and EA are present in both subgroups, individuals in the inept behavior subtype focus by definition more on their behavior and less on their bodily manifestations of anxiety. As such, the proposed self-reinforcing development system between AS and EA would become disrupted. In contrast, individuals in the noticeable symptoms subtype are likely to be hyperalert to bodily anxiety symptoms, perceiving increases in bodily arousal as signs of elevated threats for rejection or deprecation. Therefore, they may re-engage in EA, continually checking for success or failure of their attempts to reduce, modify, or suppress their anxiety symptoms, and thereby exacerbate their anxiety, resulting in a ‘*full-on downward spiral’*, as one study participant put it.

This dynamic resembles the positive feedback loop between social anxiety and EA in individuals with SAD reported by Asher, Hofmann, and Aderka, in which both constructs mediate changes in the other [10]. Our findings corroborate this notion and provide first evidence that AS may represent another important variable in this equation, especially for socially anxious individuals who are primarily concerned about displaying noticeable signs of anxiety. Their struggle seems to mirror the dynamics of panic disorder, albeit with a different focus, not fearing negative physical, but *social* consequences, as evidenced by the significantly higher mean scores on the social concerns subscale of anxiety sensitivity across the entire sample. For example, the 44-year-old Brazilian participant who reported experiencing a panic attack while reading to their colleagues during a meeting qualified as having no social anxiety according to the LSAS-SR. In such cases, treatment interventions might benefit from addressing the proposed mutually reinforcing development system between AS, EA, and social anxiety.

Based on our study’s findings, attention- and acceptance-based interventions, along with interoceptive exposure, particularly in social contexts, may hold promise as potential therapeutic approaches for individuals with the noticeable symptoms subtype of social anxiety. It can be hypothesized that addressing underlying beliefs about the perceived danger of noticeable anxiety symptoms may also be worth considering for this group. However, it is important to acknowledge that further research and clinical validation are needed to determine the efficacy and suitability of these interventions for the noticeable symptoms subtype.

Similarly, the treatment needs of the inept behavior subtype may differ, and future research could explore alternative therapeutic approaches tailored to this group’s specific characteristics. Identifying such distinctions among social anxiety subtypes may provide valuable insights for clinicians in developing more personalized and targeted treatment strategies.

Furthermore, our findings raise interesting questions about the potential mutual reinforcement between social anxiety, EA, and AS. Future quantitative studies should further investigate whether social anxiety predicts not only EA but also AS, to confirm or disconfirm our hypothesis of a mutually reinforcing development system involving these variables.

Overall, this study emphasizes the need for further research that carefully examines the heterogeneity of SAD. Investigating potential subgroups within the condition will ultimately pave the way for more targeted and effective treatments.

### Strengths and limitations

One of the study’s notable strengths is its cross-cultural nature, including participants from 21 countries and 5 different languages, which enhances the generalizability of the results across cultural and linguistic barriers. Moreover, the incorporation of participants’ perspectives stands out as an advantage, as research on social anxiety often tends to be exclusively quantitative.

However, certain limitations should be acknowledged. The use of cross-sectional correlational data limits the establishment of definitive causality. Exclusion of the "offensive" subtype restricts our findings to two subgroups, potentially missing valuable insights. While self-reported social anxiety scores may somewhat limit generalizability to clinical samples, the high mean score in social anxiety measures (81.00) partially mitigates this concern.

We acknowledge that gender data were not collected in our study. This decision aimed to minimize potential discomfort or intrusive feelings related to personal identity inquiries, aligning with the study’s primary objectives and the sensitivity of the topic.

Considering the relatively modest sample size, effect sizes were used to interpret relationships between variables. It is important to note that there might be meaningful connections between constructs that, while not statistically significant in our current analysis, warrant exploration in future studies with larger samples.

Additionally, the recruitment of participants through the ConquerSocialAnxiety.com website may have biased the selection of the sample, as the articles available on the website may appeal more to a certain group of socially anxious individuals than to others. Consequently, descriptive statistics may not accurately reflect the general population with SAD.

Due to the limited availability of studies with exact measures for AS and EA in relation to social anxiety using the LSAS-SR, we adopted conservative effect sizes from relevant studies in our power analysis. While this approach ensured cautious estimations, it should be considered in interpreting the results.

Lastly, cross-cultural studies involving qualitative data in different languages imply issues around translation. Translation is not objective and is considered an interpretive act of assigning meaning to words in the source and target languages [44]. Despite our best efforts, the cultural aspects and subjective connotations underlying the data provided in the qualitative results section may not have been captured accurately.

## Conclusions and future directions

The present study provides preliminary evidence for qualitative differences between socially anxious individuals primarily concerned about displaying noticeable signs of anxiety and those focused on behaving ineptly. The former subgroup appears to experience more pronounced challenges in the interplay between AS, EA, and social anxiety, while the latter subgroup appears to be influenced by additional constructs that may not have the same level of impact on the former. These findings emphasize the importance of improved subtyping in SAD to better understand the distinct clinical demands of each subtype. Future research efforts should continue to explore the heterogeneity of the disorder, identifying subgroups, and investigating their unique characteristics. Longitudinal studies focused on causality are warranted to examine the self-reinforcing system between AS, EA, and social anxiety over time, as supported by the substantial joint effect sizes and subtype-specific predictive properties. Such investigations will contribute to a deeper understanding of the complex interplay between these variables and offer valuable insights into developing more targeted and effective interventions for individuals with different social anxiety subtypes.

## Supporting information

S1 File Full questionnaire. English, German, Spanish, Portuguese, French Versions of Survey.
(PDF)

S2 File Statistical analyses. Power Analysis, Correlation Matrix, Descriptive Statistics, Regression.
(PDF)

S1 Dataset Quantitative data. Quantitative Data of Final Sample.
(XLSX)

S2 Dataset Qualitative data. Qualitative Sample of Final Sample.
(XLSX)
