# Supplementary material for: Differences in anxiety sensitivity and experiential avoidance between subtypes of social anxiety disorder
Source: PLoS One. 2023 Sep 15;18(9):e0290756. doi: 10.1371/journal.pone.0290756 (PMC10503767; doi:10.1371/journal.pone.0290756)
Supplement: S1 File — English, German, Spanish, Portuguese, French Versions of Survey. (PDF) [file pone.0290756.s001.pdf]

Social Anxiety Research Questionnaire (English)

This questionnaire is designed to explore the experiences of people who struggle with social anxiety. It consists of four parts and takes about 15 minutes to complete.

The last part of the questionnaire entails several open-ended questions that are intended to allow you to freely elaborate on your experiences. The more detailed your answers in this part of the survey, the more valuable they will be for our analysis.

Your answers in this survey will be anonymous. Rest assured that no personal information will be associated with any research findings.

If you are interested in getting an idea of the severity of your social anxiety, you can keep track of your scores by writing them down on a piece of paper or on your phone. You'll be reminded when relevant.

Thank you for taking the time to participate in this study.

* Required

Study Participation: Informed Consent

I have been offered to participate in this research, which will be conducted under the direction of Martin Stork, psychology student, and Mariantonia Lemos Hoyos, psychologist and professor, at Universidad EAFIT, Colombia.

The present study aims to examine the extent to which individuals with social anxiety control internal events such as thoughts, feelings and physical sensations during social situations. It also aims to identify the beliefs and attitudes of individuals with social anxiety regarding their attempts to monitor and control their internal experiences in social situations, as well as to explore any potential strategies they may employ in attempting to do so.

This study is part of the undergraduate dissertation of psychology student Martin Stork.

I have been informed that in the course of the present study I will be presented with brief questionnaires assessing different psychological aspects (anxiety, perceptions of uncomfortable feelings, coping strategies). I will then be asked to describe my unique experiences during social situations, such as my thoughts, feelings, and bodily sensations.

When I am faced with questions that I don't want to answer because they are uncomfortable, I can simply leave them blank and continue with the evaluation.

I understand that my participation in this study is voluntary and that I may withdraw at any time without penalty. Participating in this research will have no direct benefit to me. However, the results of this study may be useful for the future understanding of social anxiety disorder and, therefore, may contribute to improving its treatment.

1. Informed Consent *

- ◯ I have read this consent form and fully understand it. All questions regarding this consent and the study have been answered satisfactorily and I agree to participate in the study.
- ◯ I do not agree to participate in this study.

Part I: Liebowitz Social Anxiety Scale (LSAS-SR)

This measure assesses the way that social phobia plays a role in your life across a variety of situations.

Read each situation carefully and answer two questions about that situation. The first question asks how anxious or fearful you feel in the situation. The second question asks how often you avoid the situation.

If you come across a situation that you ordinarily do not experience, imagine "what if you were faced with that situation," and then, rate the degree to which you would fear this hypothetical situation and how often you would tend to avoid it.

Please base your ratings on the way that the situations have affected you in the last week. Fill out the following scale with the most suitable answer provided below.

As you will see, each possible answer in this questionnaire is followed by a number in parentheses, such as (1). If you are interested in knowing the severity of your social anxiety, write down the corresponding number following each answer you select (e.g., 1 - 2 - 0 - 3 - 1 - 3 - 2 - etc.). At the end of this survey, you will need these numbers if you want to interpret your scores (optional).

2. Telephoning in public.

	None (0)	Mild (1)	Moderate (2)	Severe (3)
Fear or Anxiety	○	○	○	○

3.

	Never: 0% (0)	Occasionally: 1-33% (1)	Often: 33-67% (2)	Usually: 67-100% (3)
Avoidance	○	○	○	○

4. Participating in small groups.

	None (0)	Mild (1)	Moderate (2)	Severe (3)
Fear or Anxiety	○	○	○	○

5.

	Never: 0% (0)	Occasionally: 1-33% (1)	Often: 33-67% (2)	Usually: 67-100% (3)
Avoidance	○	○	○	○

6. Eating in public places.

	None (0)	Mild (1)	Moderate (2)	Severe (3)
Fear or Anxiety	○	○	○	○

7.

	Never: 0% (0)	Occasionally: 1-33% (1)	Often: 33-67% (2)	Usually: 67-100% (3)
Avoidance	○	○	○	○

8. Drinking with others in public places.

	None (0)	Mild (1)	Moderate (2)	Severe (3)
Fear or Anxiety	○	○	○	○

9.

	Never: 0% (0)	Occasionally: 1-33% (1)	Often: 33-67% (2)	Usually: 67-100% (3)
Avoidance	○	○	○	○

10. Talking to people in authority.

	None (0)	Mild (1)	Moderate (2)	Severe (3)
Fear or Anxiety	○	○	○	○

11.

	Never: 0% (0)	Occasionally: 1-33% (1)	Often: 33-67% (2)	Usually: 67-100% (3)
Avoidance	○	○	○	○

12. Acting, performing or giving a talk in front of an audience.

	None (0)	Mild (1)	Moderate (2)	Severe (3)
Fear or Anxiety	◯	◯	◯	◯

13.

	Never: 0% (0)	Occasionally: 1-33% (1)	Often: 33-67% (2)	Usually: 67-100% (3)
Avoidance	◯	◯	◯	◯

14. Going to a party.

	None (0)	Mild (1)	Moderate (2)	Severe (3)
Fear or Anxiety	◯	◯	◯	◯

15.

	Never: 0% (0)	Occasionally: 1-33% (1)	Often: 33-67% (2)	Usually: 67-100% (3)
Avoidance	◯	◯	◯	◯

16. Working while being observed.

	None (0)	Mild (1)	Moderate (2)	Severe (3)
Fear or Anxiety	◯	◯	◯	◯

17.

	Never: 0% (0)	Occasionally: 1-33% (1)	Often: 33-67% (2)	Usually: 67-100% (3)
Avoidance	◯	◯	◯	◯

18. Writing while being observed.

	None (0)	Mild (1)	Moderate (2)	Severe (3)
Fear or Anxiety	◯	◯	◯	◯

19.

	Never: 0% (0)	Occasionally: 1-33% (1)	Often: 33-67% (2)	Usually: 67-100% (3)
Avoidance	◯	◯	◯	◯

20. Calling someone you don’t know very well.

	None (0)	Mild (1)	Moderate (2)	Severe (3)
Fear or Anxiety	◯	◯	◯	◯

21.

	Never: 0% (0)	Occasionally: 1-33% (1)	Often: 33-67% (2)	Usually: 67-100% (3)
Avoidance	◯	◯	◯	◯

22. Talking with people you don’t know very well.

	None (0)	Mild (1)	Moderate (2)	Severe (3)
Fear or Anxiety	◯	◯	◯	◯

23.

	Never: 0% (0)	Occasionally: 1-33% (1)	Often: 33-67% (2)	Usually: 67-100% (3)
Avoidance	◯	◯	◯	◯

24. Meeting strangers.

	None (0)	Mild (1)	Moderate (2)	Severe (3)
Fear or Anxiety	○	○	○	○

25.

	Never: 0% (0)	Occasionally: 1-33% (1)	Often: 33-67% (2)	Usually: 67-100% (3)
Avoidance	○	○	○	○

26. Urinating in a public bathroom.

	None (0)	Mild (1)	Moderate (2)	Severe (3)
Fear or Anxiety	○	○	○	○

27.

	Never: 0% (0)	Occasionally: 1-33% (1)	Often: 33-67% (2)	Usually: 67-100% (3)
Avoidance	○	○	○	○

28. Entering a room when others are already seated.

	None (0)	Mild (1)	Moderate (2)	Severe (3)
Fear or Anxiety	○	○	○	○

29.

	Never: 0% (0)	Occasionally: 1-33% (1)	Often: 33-67% (2)	Usually: 67-100% (3)
Avoidance	○	○	○	○

30. Being the center of attention.

	None (0)	Mild (1)	Moderate (2)	Severe (3)
Fear or Anxiety	◯	◯	◯	◯

31.

	Never: 0% (0)	Occasionally: 1-33% (1)	Often: 33-67% (2)	Usually: 67-100% (3)
Avoidance	◯	◯	◯	◯

32. Speaking up at a meeting.

	None (0)	Mild (1)	Moderate (2)	Severe (3)
Fear or Anxiety	◯	◯	◯	◯

33.

	Never: 0% (0)	Occasionally: 1-33% (1)	Often: 33-67% (2)	Usually: 67-100% (3)
Avoidance	◯	◯	◯	◯

34. Taking a test.

	None (0)	Mild (1)	Moderate (2)	Severe (3)
Fear or Anxiety	◯	◯	◯	◯

35.

	Never: 0% (0)	Occasionally: 1-33% (1)	Often: 33-67% (2)	Usually: 67-100% (3)
Avoidance	◯	◯	◯	◯

36. Expressing a disagreement or disapproval to people you don’t know very well.

	None (0)	Mild (1)	Moderate (2)	Severe (3)
Fear or Anxiety	◯	◯	◯	◯

37.

	Never: 0% (0)	Occasionally: 1-33% (1)	Often: 33-67% (2)	Usually: 67-100% (3)
Avoidance	◯	◯	◯	◯

38. Looking at people you don’t know very well in the eyes.

	None (0)	Mild (1)	Moderate (2)	Severe (3)
Fear or Anxiety	◯	◯	◯	◯

39.

	Never: 0% (0)	Occasionally: 1-33% (1)	Often: 33-67% (2)	Usually: 67-100% (3)
Avoidance	◯	◯	◯	◯

40. Giving a report to a group.

	None (0)	Mild (1)	Moderate (2)	Severe (3)
Fear or Anxiety	◯	◯	◯	◯

41.

	Never: 0% (0)	Occasionally: 1-33% (1)	Often: 33-67% (2)	Usually: 67-100% (3)
Avoidance	◯	◯	◯	◯

42. Trying to pick up someone.

	None (0)	Mild (1)	Moderate (2)	Severe (3)
Fear or Anxiety	◯	◯	◯	◯

43.

	Never: 0% (0)	Occasionally: 1-33% (1)	Often: 33-67% (2)	Usually: 67-100% (3)
Avoidance	◯	◯	◯	◯

44. Returning goods to a store.

	None (0)	Mild (1)	Moderate (2)	Severe (3)
Fear or Anxiety	◯	◯	◯	◯

45.

	Never: 0% (0)	Occasionally: 1-33% (1)	Often: 33-67% (2)	Usually: 67-100% (3)
Avoidance	◯	◯	◯	◯

46. Giving a party.

	None (0)	Mild (1)	Moderate (2)	Severe (3)
Fear or Anxiety	◯	◯	◯	◯

47.

	Never: 0% (0)	Occasionally: 1-33% (1)	Often: 33-67% (2)	Usually: 67-100% (3)
Avoidance	◯	◯	◯	◯

48. Resisting a high pressure salesperson.

	None (0)	Mild (1)	Moderate (2)	Severe (3)
Fear or Anxiety	◯	◯	◯	◯

49.

	Never: 0% (0)	Occasionally: 1-33% (1)	Often: 33-67% (2)	Usually: 67-100% (3)
Avoidance	◯	◯	◯	◯

Part II: Acceptance and Action Questionnaire–II (AAQ-II)

You have just completed the most extensive part of the survey. Please continue, the next parts are much shorter!

Also, from now on, you do not need to keep track of the numbers in parentheses behind each answer (this only applied to the first questionnaire).

Below you will find a list of statements. Please rate how true each statement is for you by selecting a number next to it. Use the scale below to make your choice.

1 = Never true
2 = Very seldom true
3 = Seldom true
4 = Sometimes true
5 = Frequently true
6 = Almost always true
7 = Always true

50.

	Never true (1)	Very seldom true (2)	Seldom true (3)	Sometimes true (4)	Frequently true (5)	Almost always true (6)	Always true (7)
My painful experiences and memories make it difficult for me to live a life that I would value.	◯	◯	◯	◯	◯	◯	◯

51.

	Never true (1)	Very seldom true (2)	Seldom true (3)	Sometimes true (4)	Frequently true (5)	Almost always true (6)	Always true (7)
I'm afraid of my feelings.	◯	◯	◯	◯	◯	◯	◯

52.

	Never true (1)	Very seldom true (2)	Seldom true (3)	Sometimes true (4)	Frequently true (5)	Almost always true (6)	Always true (7)
I worry about not being able to control my worries and feelings.	◯	◯	◯	◯	◯	◯	◯

53.

	Never true (1)	Very seldom true (2)	Seldom true (3)	Sometimes true (4)	Frequently true (5)	Almost always true (6)	Always true (7)
My painful memories prevent me from having a fulfilling life.	○	○	○	○	○	○	○

54.

	Never true (1)	Very seldom true (2)	Seldom true (3)	Sometimes true (4)	Frequently true (5)	Almost always true (6)	Always true (7)
Emotions cause problems in my life.	○	○	○	○	○	○	○

55.

	Never true (1)	Very seldom true (2)	Seldom true (3)	Sometimes true (4)	Frequently true (5)	Almost always true (6)	Always true (7)
It seems like most people are handling their lives better than I am.	○	○	○	○	○	○	○

56.

	Never true (1)	Very seldom true (2)	Seldom true (3)	Sometimes true (4)	Frequently true (5)	Almost always true (6)	Always true (7)
Worries get in the way of my success.	○	○	○	○	○	○	○

Part III: Anxiety Sensitivity Index 3 (ASI-III)

This is the last questionnaire. When you are done with this part, there are only a few short open questions waiting for you and you are done!

Again, you do not need to keep track of your scores here by writing them down.

Please rate each item by selecting one of the five answers for each question. Please answer each statement by circling the number that best applies to you.

0 = Very little
1 = A little
2 = Some
3 = Much
4 = Very much

57.

	Very little (0)	A little (1)	Some (2)	Much (3)	Very much (4)
It is important for me not to appear nervous.	○	○	○	○	○

58.

	Very little (0)	A little (1)	Some (2)	Much (3)	Very much (4)
When I cannot keep my mind on a task, I worry that I might be going crazy.	○	○	○	○	○

59.

	Very little (0)	A little (1)	Some (2)	Much (3)	Very much (4)
It scares me when my heart beats rapidly.	○	○	○	○	○

60.

	Very little (0)	A little (1)	Some (2)	Much (3)	Very much (4)
When my stomach is upset, I worry that I might be seriously ill.	◯	◯	◯	◯	◯

61.

	Very little (0)	A little (1)	Some (2)	Much (3)	Very much (4)
It scares me when I am unable to keep my mind on a task.	◯	◯	◯	◯	◯

62.

	Very little (0)	A little (1)	Some (2)	Much (3)	Very much (4)
When I tremble in the presence of others, I fear what people might think of me.	◯	◯	◯	◯	◯

63.

	Very little (0)	A little (1)	Some (2)	Much (3)	Very much (4)
When my chest feels tight, I get scared that I won’t be able to breathe properly.	◯	◯	◯	◯	◯

64.

	Very little (0)	A little (1)	Some (2)	Much (3)	Very much (4)
When I feel pain in my chest, I worry that I'm going to have a heart attack.	◯	◯	◯	◯	◯

65.

	Very little (0)	A little (1)	Some (2)	Much (3)	Very much (4)
I worry that other people will notice my anxiety.	◯	◯	◯	◯	◯

66.

	Very little (0)	A little (1)	Some (2)	Much (3)	Very much (4)
When I feel "spacey" or spaced out I worry that I may be mentally ill.	◯	◯	◯	◯	◯

67.

	Very little (0)	A little (1)	Some (2)	Much (3)	Very much (4)
It scares me when I blush in front of people.	◯	◯	◯	◯	◯

68.

	Very little (0)	A little (1)	Some (2)	Much (3)	Very much (4)
When I notice my heart skipping a beat, I worry that there is something seriously wrong with me.	◯	◯	◯	◯	◯

69.

	Very little (0)	A little (1)	Some (2)	Much (3)	Very much (4)
When I begin to sweat in a social situation, I fear people will think negatively of me.	◯	◯	◯	◯	◯

70.

	Very little (0)	A little (1)	Some (2)	Much (3)	Very much (4)
When my thoughts seem to speed up, I worry that I might be going crazy.	◯	◯	◯	◯	◯

71.

	Very little (0)	A little (1)	Some (2)	Much (3)	Very much (4)
When my throat feels tight, I worry that I could choke to death.	◯	◯	◯	◯	◯

72.

	Very little (0)	A little (1)	Some (2)	Much (3)	Very much (4)
When I have trouble thinking clearly, I worry that there is something wrong with me.	◯	◯	◯	◯	◯

73.

	Very little (0)	A little (1)	Some (2)	Much (3)	Very much (4)
I think it would be horrible for me to faint in public.	◯	◯	◯	◯	◯

74.

	Very little (0)	A little (1)	Some (2)	Much (3)	Very much (4)
When my mind goes blank, I worry there is something terribly wrong with me.	◯	◯	◯	◯	◯

Part IV: Open-Ended Questions

Almost done! This section evaluates your experiences with uncomfortable and intrusive thoughts, feelings, and physical reactions.

There is no right or wrong way to comprehend and answer these questions. The idea here is for you to elaborate freely on your experiences, without thinking too much about whether you are doing it right or wrong.

The more detailed your answers are, the more valuable they will be to our analysis. Thank you for taking the time.

75. When you find yourself in a social situation you perceive as stressful, do you commonly experience any of the following?

    -Unwanted or uncomfortable thoughts that seem to come out of nowhere.

    -Unwanted or uncomfortable feelings or emotions.

    -Unwanted or uncomfortable bodily reactions or physical manifestations of anxiety.

    If so, please name and describe these phenomena.

76. How does it affect you when you notice unwanted or uncomfortable thoughts, feelings, or bodily reactions arise during a social situation?

77. Is there a specific type of social situation in which you experience unwanted or uncomfortable thoughts, feelings, or bodily reactions as particularly upsetting?

78. When you notice unwanted or uncomfortable thoughts, feelings or bodily reactions arise during a social situation, how do you usually react?

79. How does the way you respond to unwanted or uncomfortable thoughts, feelings, or bodily reactions during social situations affect your anxiety or nervousness?

80. Select the option that best describes your case.

- ◯ I experience fear or anxiety in virtually all social situations.
- ◯ I experience fear or anxiety in a considerable number of social situations.
- ◯ I experience fear or anxiety in one or very few social situations.
- ◯ None of the above

81. Select the option that best describes your case.

- ◯ In social situations, I am mainly concerned about behaving in an inept or foolish manner (such as saying something out of place or something that may be perceived as 'stupid', etc.).
- ◯ In social situations, I am mainly concerned about displaying observable signs of anxiety (such as excessive sweating, blushing, a shaky voice, etc.).
- ◯ In social situations, I am mainly concerned about the possibility of offending others (such as saying something that might hurt their feelings, etc.).
- ◯ None of the above

82. Please state your nationality.

83. Please state your age.

This content is neither created nor endorsed by Microsoft. The data you submit will be sent to the form owner.

Microsoft Forms

Forschungsfragebogen zu sozialer Angst (Deutsch)

Dieser Fragebogen wurde entwickelt, um die Erfahrungen von Menschen zu erforschen, die mit sozialer Angst zu kämpfen haben. Er besteht aus vier Teilen und nimmt etwa 15 Minuten in Anspruch.

Der letzte Teil des Fragebogens besteht aus mehreren offenen Fragen, die Ihnen die Möglichkeit geben sollen, Ihre Erfahrungen frei zu erläutern. Je ausführlicher Ihre Antworten in diesem Teil der Umfrage sind, desto wertvoller sind diese für unsere Analyse.

Ihre Antworten in dieser Umfrage bleiben anonym. Seien Sie versichert, dass keine persönlichen Informationen mit den Forschungsergebnissen in Verbindung gebracht werden.

Wenn Sie sich ein Bild vom Schweregrad Ihrer sozialen Angst machen möchten, können Sie Ihre Werte auf einem Blatt Papier oder auf Ihrem Handy notieren. Sie werden dann daran erinnert, wenn es relevant ist.

Vielen Dank, dass Sie sich die Zeit nehmen, an dieser Studie teilzunehmen.

* Required

Teilnahme an der Studie: Informierte Zustimmung

Mir wurde angeboten, an dieser Studie teilzunehmen, die unter der Leitung von Martin Stork, Psychologiestudent, und Mariantonia Lemos Hoyos, Psychologin und Professorin, an der Universidad EAFIT, Kolumbien, durchgeführt wird.

Mit der vorliegenden Studie soll untersucht werden, inwieweit Personen mit sozialer Angst interne Ereignisse wie Gedanken, Gefühle und körperliche Empfindungen während sozialer Situationen kontrollieren. Sie zielt auch darauf ab, die Überzeugungen und Einstellungen von Personen mit sozialer Angst in Bezug auf ihre Versuche, ihre inneren Erlebnisse in sozialen Situationen zu überwachen und zu kontrollieren, zu ermitteln und mögliche Strategien zu erforschen, die sie bei dem Versuch, dies zu tun, einsetzen.

Diese Studie ist Teil der Dissertation des Psychologiestudenten Martin Stork.

Ich wurde darüber informiert, dass mir im Rahmen dieser Studie kurze Fragebögen vorgelegt werden, die verschiedene psychologische Aspekte (Angst, Wahrnehmung unangenehmer Gefühle, Bewältigungsstrategien) erfassen. Anschließend werde ich gebeten, meine besonderen Erfahrungen in sozialen Situationen zu beschreiben, wie z.B. meine Gedanken, Gefühle und Körperempfindungen.

Wenn ich mit Fragen konfrontiert werde, die ich nicht beantworten möchte, weil sie mir unangenehm sind, kann ich sie einfach auslassen und mit der Auswertung fortfahren.

Mir ist bewusst, dass meine Teilnahme an dieser Studie freiwillig ist und dass ich sie jederzeit abbrechen kann, ohne dass mir dadurch Nachteile entstehen. Die Teilnahme an dieser Studie hat keinen direkten Nutzen für mich. Die Ergebnisse dieser Studie können jedoch für das zukünftige Verständnis der sozialen Angststörung nützlich sein und somit zur Verbesserung ihrer Behandlung beitragen.

1. Einverständniserklärung *

- ◯ Ich habe diese Einverständniserklärung gelesen und verstehe sie vollständig. Alle Fragen zu dieser Einverständniserklärung und zur Studie wurden zufriedenstellend beantwortet und ich bin damit einverstanden, an der Studie teilzunehmen.
- ◯ Ich bin nicht damit einverstanden, an dieser Studie teilzunehmen.

Teil I: Liebowitz soziale Angst Skala (LSAS)

Dieser Fragebogen bewertet die Bedeutung der sozialen Phobie in Ihrem Leben in einer Reihe von Situationen.

Lesen Sie jede Situation sorgfältig durch und beantworten Sie zwei Fragen zu dieser Situation. Die erste Frage bezieht sich darauf, wie besorgt oder ängstlich Sie sich in der Situation fühlen. Die zweite Frage bezieht sich darauf, wie oft Sie die Situation vermeiden.

Wenn Sie auf eine Situation stoßen, die Sie normalerweise nicht erleben, stellen Sie sich vor, "was wäre, wenn Sie mit dieser Situation konfrontiert wären", und bewerten Sie dann, wie sehr Sie sich vor dieser hypothetischen Situation fürchten würden und wie oft Sie dazu neigen würden, sie zu vermeiden.

Bitte stützen Sie sich bei Ihren Bewertungen auf die Art und Weise, wie sich die Situationen in der letzten Woche auf Sie ausgewirkt haben. Füllen Sie die folgende Skala aus und geben Sie die am besten geeigneten Antworten an.

Wie Sie sehen werden, folgt auf jede mögliche Antwort in diesem Fragebogen eine Zahl in Klammern, wie z.B. (1). Wenn Sie wissen möchten, wie stark Ihre soziale Angst ausgeprägt ist, notieren Sie die entsprechende Zahl nach jeder Antwort, die Sie ausgewählt haben (z.B. 1 - 2 - 0 - 3 - 1 - 3 - 2 - usw.). Am Ende dieser Umfrage benötigen Sie diese Zahlen, wenn Sie Ihre Ergebnisse interpretieren möchten (optional).

2. In der Öffentlichkeit telefonieren.

	Keine (0)	Gering (1)	Mäßig (2)	Stark (3)
Angst	○	○	○	○

3.

	Nie: 0% (0)	Selten: 1-33% (1)	Häufig: 33-67% (2)	Fast immer: 67-100% (3)
Vermeidung	○	○	○	○

4. Teilnahme an einer Aktivität in kleiner Gruppe.

	Keine (0)	Gering (1)	Mäßig (2)	Stark (3)
Angst	○	○	○	○

5.

	Nie: 0% (0)	Selten: 1-33% (1)	Häufig: 33-67% (2)	Fast immer: 67-100% (3)
Vermeidung	○	○	○	○

6. In der Öffentlichkeit essen.

	Keine (0)	Gering (1)	Mäßig (2)	Stark (3)
Angst	◯	◯	◯	◯

7.

	Nie: 0% (0)	Selten: 1-33% (1)	Häufig: 33-67% (2)	Fast immer: 67-100% (3)
Vermeidung	◯	◯	◯	◯

8. In der Öffentlichkeit trinken.

	Keine (0)	Gering (1)	Mäßig (2)	Stark (3)
Angst	◯	◯	◯	◯

9.

	Nie: 0% (0)	Selten: 1-33% (1)	Häufig: 33-67% (2)	Fast immer: 67-100% (3)
Vermeidung	◯	◯	◯	◯

10. Mit Vorgesetzten oder Autoritätspersonen sprechen.

	Keine (0)	Gering (1)	Mäßig (2)	Stark (3)
Angst	◯	◯	◯	◯

11.

	Nie: 0% (0)	Selten: 1-33% (1)	Häufig: 33-67% (2)	Fast immer: 67-100% (3)
Vermeidung	◯	◯	◯	◯

12. Vor Publikum auftreten, handeln oder sprechen.

	Keine (0)	Gering (1)	Mäßig (2)	Stark (3)
Angst	○	○	○	○

13.

	Nie: 0% (0)	Selten: 1-33% (1)	Häufig: 33-67% (2)	Fast immer: 67-100% (3)
Vermeidung	○	○	○	○

14. Zu einem Fest, einer Party gehen.

	Keine (0)	Gering (1)	Mäßig (2)	Stark (3)
Angst	○	○	○	○

15.

	Nie: 0% (0)	Selten: 1-33% (1)	Häufig: 33-67% (2)	Fast immer: 67-100% (3)
Vermeidung	○	○	○	○

16. Bei der Arbeit beobachtet werden.

	Keine (0)	Gering (1)	Mäßig (2)	Stark (3)
Angst	○	○	○	○

17.

	Nie: 0% (0)	Selten: 1-33% (1)	Häufig: 33-67% (2)	Fast immer: 67-100% (3)
Vermeidung	○	○	○	○

18. Beim Schreiben beobachtet werden.

	Keine (0)	Gering (1)	Mäßig (2)	Stark (3)
Angst	◯	◯	◯	◯

19.

	Nie: 0% (0)	Selten: 1-33% (1)	Häufig: 33-67% (2)	Fast immer: 67-100% (3)
Vermeidung	◯	◯	◯	◯

20. Mit jemandem telefonieren, den man kaum kennt.

	Keine (0)	Gering (1)	Mäßig (2)	Stark (3)
Angst	◯	◯	◯	◯

21.

	Nie: 0% (0)	Selten: 1-33% (1)	Häufig: 33-67% (2)	Fast immer: 67-100% (3)
Vermeidung	◯	◯	◯	◯

22. Mit jemandem sprechen, den man kaum kennt.

	Keine (0)	Gering (1)	Mäßig (2)	Stark (3)
Angst	◯	◯	◯	◯

23.

	Nie: 0% (0)	Selten: 1-33% (1)	Häufig: 33-67% (2)	Fast immer: 67-100% (3)
Vermeidung	◯	◯	◯	◯

24. Mit Fremden zusammentreffen.

	Keine (0)	Gering (1)	Mäßig (2)	Stark (3)
Angst	◯	◯	◯	◯

25.

	Nie: 0% (0)	Selten: 1-33% (1)	Häufig: 33-67% (2)	Fast immer: 67-100% (3)
Vermeidung	◯	◯	◯	◯

26. Aufsuchen einer öffentlichen Toilette.

	Keine (0)	Gering (1)	Mäßig (2)	Stark (3)
Angst	◯	◯	◯	◯

27.

	Nie: 0% (0)	Selten: 1-33% (1)	Häufig: 33-67% (2)	Fast immer: 67-100% (3)
Vermeidung	◯	◯	◯	◯

28. Einen Raum betreten, in dem andere bereits sitzen.

	Keine (0)	Gering (1)	Mäßig (2)	Stark (3)
Angst	◯	◯	◯	◯

29.

	Nie: 0% (0)	Selten: 1-33% (1)	Häufig: 33-67% (2)	Fast immer: 67-100% (3)
Vermeidung	◯	◯	◯	◯

30. Im Mittelpunkt der Aufmerksamkeit stehen.

	Keine (0)	Gering (1)	Mäßig (2)	Stark (3)
Angst	◯	◯	◯	◯

31.

	Nie: 0% (0)	Selten: 1-33% (1)	Häufig: 33-67% (2)	Fast immer: 67-100% (3)
Vermeidung	◯	◯	◯	◯

32. Ohne Vorbereitung auf Veranstaltungen sprechen.

	Keine (0)	Gering (1)	Mäßig (2)	Stark (3)
Angst	◯	◯	◯	◯

33.

	Nie: 0% (0)	Selten: 1-33% (1)	Häufig: 33-67% (2)	Fast immer: 67-100% (3)
Vermeidung	◯	◯	◯	◯

34. An einem Test teilnehmen.

	Keine (0)	Gering (1)	Mäßig (2)	Stark (3)
Angst	◯	◯	◯	◯

35.

	Nie: 0% (0)	Selten: 1-33% (1)	Häufig: 33-67% (2)	Fast immer: 67-100% (3)
Vermeidung	◯	◯	◯	◯

36. Gegenüber jemandem, den man kaum kennt, seine fehlende Zustimmung oder Anerkennung äußern.

	Keine (0)	Gering (1)	Mäßig (2)	Stark (3)
Angst	◯	◯	◯	◯

37.

	Nie: 0% (0)	Selten: 1-33% (1)	Häufig: 33-67% (2)	Fast immer: 67-100% (3)
Vermeidung	◯	◯	◯	◯

38. Jemandem, den man weniger kennt, direkt in die Augen schauen.

	Keine (0)	Gering (1)	Mäßig (2)	Stark (3)
Angst	◯	◯	◯	◯

39.

	Nie: 0% (0)	Selten: 1-33% (1)	Häufig: 33-67% (2)	Fast immer: 67-100% (3)
Vermeidung	◯	◯	◯	◯

40. Vor einer Gruppe einen vorbereiteten mündlichen Bericht geben.

	Keine (0)	Gering (1)	Mäßig (2)	Stark (3)
Angst	◯	◯	◯	◯

41.

	Nie: 0% (0)	Selten: 1-33% (1)	Häufig: 33-67% (2)	Fast immer: 67-100% (3)
Vermeidung	◯	◯	◯	◯

42. Eine Liebes- oder Intimbeziehung aufnehmen.

	Keine (0)	Gering (1)	Mäßig (2)	Stark (3)
Angst	◯	◯	◯	◯

43.

	Nie: 0% (0)	Selten: 1-33% (1)	Häufig: 33-67% (2)	Fast immer: 67-100% (3)
Vermeidung	◯	◯	◯	◯

44. Waren in einem Geschäft umtauschen.

	Keine (0)	Gering (1)	Mäßig (2)	Stark (3)
Angst	◯	◯	◯	◯

45.

	Nie: 0% (0)	Selten: 1-33% (1)	Häufig: 33-67% (2)	Fast immer: 67-100% (3)
Vermeidung	◯	◯	◯	◯

46. Ein Fest, eine Party geben.

	Keine (0)	Gering (1)	Mäßig (2)	Stark (3)
Angst	◯	◯	◯	◯

47.

	Nie: 0% (0)	Selten: 1-33% (1)	Häufig: 33-67% (2)	Fast immer: 67-100% (3)
Vermeidung	◯	◯	◯	◯

48. Dem hohen Druck eines Verkäufers widerstehen.

	Keine (0)	Gering (1)	Mäßig (2)	Stark (3)
Angst	◯	◯	◯	◯

49.

	Nie: 0% (0)	Selten: 1-33% (1)	Häufig: 33-67% (2)	Fast immer: 67-100% (3)
Vermeidung	◯	◯	◯	◯

Teil II: Fragebogen zu Akzeptanz und Handeln II (FAH-II)

Sie haben soeben den umfangreichsten Teil des Fragebogens ausgefüllt. Bitte fahren Sie fort, die nächsten Teile sind viel kürzer!

Außerdem brauchen Sie von nun an die Zahlen in Klammern hinter jeder Antwort nicht mehr zu notieren (dies galt nur für den ersten Fragebogen).

Sie finden unten eine Liste mit Aussagen. Bitte geben Sie an, wie wahr jede Aussage für
Sie ist, indem Sie eine der nebenstehenden Nummern auswählen. Nutzen Sie die folgende Skala für Ihre Auswahl.

1 = Niemals wahr
2 = Sehr selten wahr
3 = Selten wahr
4 = Manchmal wahr
5 = Häufig wahr
6 = Fast immer wahr
7 = Immer wahr

50.

	Niemals wahr (1)	Sehr selten wahr (2)	Selten wahr (3)	Manchmal wahr (4)	Häufig wahr (5)	Fast immer wahr (6)	Immer wahr (7)
Meine schmerzlichen Erfahrungen und Erinnerungen machen es mir schwer, ein Leben zu leben, das ich wertschätzen würde.	○	○	○	○	○	○	○

51.

	Niemals wahr (1)	Sehr selten wahr (2)	Selten wahr (3)	Manchmal wahr (4)	Häufig wahr (5)	Fast immer wahr (6)	Immer wahr (7)
Ich habe Angst vor meinen Gefühlen.	○	○	○	○	○	○	○

52.

	Niemals wahr (1)	Sehr selten wahr (2)	Selten wahr (3)	Manchmal wahr (4)	Häufig wahr (5)	Fast immer wahr (6)	Immer wahr (7)
Ich sorge mich darum, nicht fähig zu sein, meine Sorgen und Gefühle zu kontrollieren.	○	○	○	○	○	○	○

53.

	Niemals wahr (1)	Sehr selten wahr (2)	Selten wahr (3)	Manchmal wahr (4)	Häufig wahr (5)	Fast immer wahr (6)	Immer wahr (7)
Meine schmerzlichen Erinnerungen halten mich davon ab, ein erfülltes Leben zu haben.	◯	◯	◯	◯	◯	◯	◯

54.

	Niemals wahr (1)	Sehr selten wahr (2)	Selten wahr (3)	Manchmal wahr (4)	Häufig wahr (5)	Fast immer wahr (6)	Immer wahr (7)
Emotionen verursachen Probleme in meinem Leben.	◯	◯	◯	◯	◯	◯	◯

55.

	Niemals wahr (1)	Sehr selten wahr (2)	Selten wahr (3)	Manchmal wahr (4)	Häufig wahr (5)	Fast immer wahr (6)	Immer wahr (7)
Es scheint, als ob die meisten Leute ihr Leben besser bewältigen als ich.	◯	◯	◯	◯	◯	◯	◯

56.

	Niemals wahr (1)	Sehr selten wahr (2)	Selten wahr (3)	Manchmal wahr (4)	Häufig wahr (5)	Fast immer wahr (6)	Immer wahr (7)
Sorgen stellen sich meinem Erfolg in den Weg.	◯	◯	◯	◯	◯	◯	◯

Teil III: Angstsensitivitätsindex-3 (ASI-3)

Dies ist der letzte Fragebogen. Wenn Sie mit diesem Teil fertig sind, warten nur noch ein paar kurze offene Fragen auf Sie und Sie sind fertig!

Auch hier brauchen Sie Ihre Punkte nicht aufzuschreiben.

Bitte wählen Sie die Zahl aus, die den Grad Ihrer Zustimmung zu einer Aussage am besten ausdrückt.

Sollte eine Aussage Inhalte thematisieren, die Sie nicht erlebt haben, (z. B. "Es macht mir Angst, wenn ich vor anderen Menschen erröte" wenn Sie noch nie vor anderen errötet sind), antworten Sie bitte gemäß der Erwartung, wie Sie sich bei einer solchen Erfahrung fühlen würden. Andernfalls beantworten Sie die Aussage auf Basis Ihrer eigenen Erfahrung.

Bitte achten Sie darauf, bei jeder Aussage nur ein Kreuz zu machen und möglichst alle Aussagen zu beantworten.

0 = Stimme gar nicht zu
1 = Stimme wenig zu
2 = Stimme teils teils zu
3 = Stimme ziemlich zu
4 = Stimme völlig zu

57.

	Stimme gar nicht zu (0)	Stimme wenig zu (1)	Stimme teils teils zu (2)	Stimme ziemlich zu (3)	Stimme völlig zu (4)
Es ist mir wichtig, nicht nervös zu erscheinen.	◯	◯	◯	◯	◯

58.

	Stimme gar nicht zu (0)	Stimme wenig zu (1)	Stimme teils teils zu (2)	Stimme ziemlich zu (3)	Stimme völlig zu (4)
Wenn ich mich nicht auf eine Aufgabe konzentrieren kann, befürchte ich, verrückt zu werden.	◯	◯	◯	◯	◯

59.

	Stimme gar nicht zu (0)	Stimme wenig zu (1)	Stimme teils teils zu (2)	Stimme ziemlich zu (3)	Stimme völlig zu (4)
Es macht mir Angst, wenn ich starkes Herzklopfen verspüre.	◯	◯	◯	◯	◯

60.

	Stimme gar nicht zu (0)	Stimme wenig zu (1)	Stimme teils teils zu (2)	Stimme ziemlich zu (3)	Stimme völlig zu (4)
Wenn ich mir den Magen verdorben habe, befürchte ich, dass ich ernsthaft krank bin.	◯	◯	◯	◯	◯

61.

	Stimme gar nicht zu (0)	Stimme wenig zu (1)	Stimme teils teils zu (2)	Stimme ziemlich zu (3)	Stimme völlig zu (4)
Es macht mir Angst, wenn ich mich nicht auf eine Aufgabe konzentrieren kann.	◯	◯	◯	◯	◯

62.

	Stimme gar nicht zu (0)	Stimme wenig zu (1)	Stimme teils teils zu (2)	Stimme ziemlich zu (3)	Stimme völlig zu (4)
Wenn ich in Gegenwart anderer zittere, fürchte ich, was diese Personen von mir denken.	◯	◯	◯	◯	◯

63.

	Stimme gar nicht zu (0)	Stimme wenig zu (1)	Stimme teils teils zu (2)	Stimme ziemlich zu (3)	Stimme völlig zu (4)
Wenn ich ein Beklemmungsgefühl in der Brust habe, befürchte ich, dass ich nicht mehr richtig atmen kann.	◯	◯	◯	◯	◯

64.

	Stimme gar nicht zu (0)	Stimme wenig zu (1)	Stimme teils teils zu (2)	Stimme ziemlich zu (3)	Stimme völlig zu (4)
Wenn ich Schmerzen in meiner Brust habe, befürchte ich, einen Herzinfarkt zu bekommen.	◯	◯	◯	◯	◯

65.

	Stimme gar nicht zu (0)	Stimme wenig zu (1)	Stimme teils teils zu (2)	Stimme ziemlich zu (3)	Stimme völlig zu (4)
Es macht mir Sorgen, dass andere Personen meine Angst bemerken könnten.	◯	◯	◯	◯	◯

66.

	Stimme gar nicht zu (0)	Stimme wenig zu (1)	Stimme teils teils zu (2)	Stimme ziemlich zu (3)	Stimme völlig zu (4)
Wenn ich das Gefühl habe neben mir zu stehen, befürchte ich, dass ich seelisch krank bin.	◯	◯	◯	◯	◯

67.

	Stimme gar nicht zu (0)	Stimme wenig zu (1)	Stimme teils teils zu (2)	Stimme ziemlich zu (3)	Stimme völlig zu (4)
Es macht mir Angst, wenn ich vor anderen Menschen erröte.	◯	◯	◯	◯	◯

68.

	Stimme gar nicht zu (0)	Stimme wenig zu (1)	Stimme teils teils zu (2)	Stimme ziemlich zu (3)	Stimme völlig zu (4)
Wenn ich bemerke, dass mein Herz für einen Moment aussetzt, befürchte ich, dass etwas mit mir nicht stimmt.	◯	◯	◯	◯	◯

69.

	Stimme gar nicht zu (0)	Stimme wenig zu (1)	Stimme teils teils zu (2)	Stimme ziemlich zu (3)	Stimme völlig zu (4)
Wenn ich in Anwesenheit anderer anfange zu schwitzen, fürchte ich, dass sie negativ über mich denken.	◯	◯	◯	◯	◯

70.

	Stimme gar nicht zu (0)	Stimme wenig zu (1)	Stimme teils teils zu (2)	Stimme ziemlich zu (3)	Stimme völlig zu (4)
Wenn sich meine Gedanken beschleunigen, fürchte ich, dass ich verrückt werde.	◯	◯	◯	◯	◯

71.

	Stimme gar nicht zu (0)	Stimme wenig zu (1)	Stimme teils teils zu (2)	Stimme ziemlich zu (3)	Stimme völlig zu (4)
Wenn sich meine Kehle eng anfühlt, habe ich Angst, dass ich ersticken könnte.	◯	◯	◯	◯	◯

72.

	Stimme gar nicht zu (0)	Stimme wenig zu (1)	Stimme teils teils zu (2)	Stimme ziemlich zu (3)	Stimme völlig zu (4)
Wenn ich Schwierigkeiten habe, klar zu denken, befürchte ich, dass etwas mit mir nicht stimmt.	◯	◯	◯	◯	◯

73.

	Stimme gar nicht zu (0)	Stimme wenig zu (1)	Stimme teils teils zu (2)	Stimme ziemlich zu (3)	Stimme völlig zu (4)
Ich glaube, dass es schrecklich für mich wäre, in der Öffentlichkeit in Ohnmacht zu fallen	◯	◯	◯	◯	◯

74.

	Stimme gar nicht zu (0)	Stimme wenig zu (1)	Stimme teils teils zu (2)	Stimme ziemlich zu (3)	Stimme völlig zu (4)
Wenn ich einen "Blackout" habe, befürchte ich, dass mit mir etwas ganz und gar nicht stimmt.	◯	◯	◯	◯	◯

Teil IV: Offene Fragen

Fast fertig! In diesem Abschnitt werden Ihre Erfahrungen mit unangenehmen und aufdringlichen Gedanken, Gefühlen und körperlichen Reaktionen bewertet.

Es gibt keine richtige oder falsche Art, diese Fragen zu verstehen und zu beantworten. Die Idee ist, dass Sie frei über Ihre Erfahrungen sprechen, ohne zu sehr darüber nachzudenken, ob Sie es richtig oder falsch machen.

Je detaillierter Ihre Antworten sind, desto wertvoller sind diese für unsere Analyse. Vielen Dank, dass Sie sich die Zeit nehmen.

75. Wenn Sie sich in einer sozialen Situation befinden, die Sie als stressig empfinden, erleben Sie dann häufig eines der folgenden Dinge?

    -Unerwünschte oder unangenehme Gedanken, die aus dem Nichts zu kommen scheinen.

    -Unerwünschte oder unangenehme Gefühle oder Emotionen.

    -Unerwünschte oder unangenehme körperliche Reaktionen oder körperliche Anzeichen von Angst.

    Wenn ja, nennen und beschreiben Sie bitte diese Phänomene.

76. Welche Auswirkungen hat es auf Sie, wenn Sie bemerken, dass bei Ihnen in einer sozialen Situation unerwünschte oder unangenehme Gedanken, Gefühle oder körperliche Reaktionen auftreten?

77. Gibt es eine bestimmte Art von sozialer Situation, in der Sie unerwünschte oder unangenehme Gedanken, Gefühle oder körperliche Reaktionen als besonders belastend empfinden?

78. Wenn Sie bemerken, dass bei Ihnen in einer sozialen Situation unerwünschte oder unangenehme Gedanken, Gefühle oder körperliche Reaktionen auftreten, wie reagieren Sie dann normalerweise?

79. Wie beeinflusst die Art und Weise, wie Sie auf unerwünschte oder unangenehme Gedanken, Gefühle oder körperliche Reaktionen in sozialen Situationen reagieren, Ihre Angst oder Nervosität?

80. Wählen Sie die Option, die Ihren Fall am besten beschreibt.

- Ich empfinde in praktisch allen sozialen Situationen Angst oder Beklemmung.
- Ich empfinde in einer beträchtlichen Anzahl von sozialen Situationen Angst oder Beklemmung.
- Ich empfinde Angst oder Beklemmung in einer bestimmten oder sehr wenigen sozialen Situationen.
- Keine der oben genannten Möglichkeiten.

81. Wählen Sie die Option, die Ihren Fall am besten beschreibt.

- In sozialen Situationen mache ich mir vor allem Sorgen darüber, dass ich mich ungeschickt oder töricht verhalte (z. B. etwas Unangebrachtes sage oder etwas, das als "dumm" empfunden werden könnte usw.).
- In sozialen Situationen bin ich vor allem besorgt darüber, erkennbare Anzeichen von Angst zu zeigen (wie übermäßiges Schwitzen, Erröten, eine zittrige Stimme usw.).
- In sozialen Situationen mache ich mir vor allem Sorgen über die Möglichkeit, andere beleidigen zu können (z. B. etwas zu sagen, das ihre Gefühle verletzen könnte usw.).
- Keine der oben genannten Möglichkeiten

82. Bitte geben Sie Ihre Nationalität an.

83. Bitte geben Sie Ihr Alter an.

This content is neither created nor endorsed by Microsoft. The data you submit will be sent to the form owner.

Microsoft Forms

Cuestionario de Investigación de Ansiedad Social (Español)

Este cuestionario está diseñado para explorar las experiencias de personas que padecen ansiedad social. Consta de cuatro partes y requiere unos 15 minutos para completarlo.

La última parte del cuestionario está compuesta por varias preguntas abiertas que pretenden permitirle elaborar libremente sobre sus experiencias. Cuanto más detalladas sean sus respuestas en esta parte de la encuesta, más valiosas serán para nuestro análisis.

Sus respuestas en esta encuesta serán anónimas. Puede estar seguro de que no se asociará ninguna información personal con los resultados de la investigación.

Si le interesa hacerse una idea de la gravedad de su ansiedad social, puede llevar un registro de sus puntuaciones anotándolas en un papel o en su teléfono. Se le recordará cuando sea pertinente.

Gracias por dedicar su tiempo a participar en este estudio.

* Required

Participación en el estudio: Consentimiento informado

Me han ofrecido participar en esta investigación, que se llevará a cabo bajo la dirección de Martin Stork, estudiante de psicología, y Mariantonia Lemos Hoyos, psicóloga y profesora, en la Universidad EAFIT, Colombia.

El presente estudio pretende examinar hasta qué punto los individuos con ansiedad social controlan los acontecimientos internos como los pensamientos, los sentimientos y las sensaciones físicas durante las situaciones sociales. También pretende identificar las creencias y actitudes de los individuos con ansiedad social respecto a sus intentos de vigilar y controlar sus experiencias internas en situaciones sociales, así como explorar las posibles estrategias que emplean para intentar hacerlo.

Este estudio forma parte del trabajo de grado del estudiante de psicología Martin Stork.

Se me ha informado de que en el transcurso del presente estudio se me presentarán breves cuestionarios en los que se evaluarán diferentes aspectos psicológicos (ansiedad, percepción de sentimientos incómodos, estrategias de afrontamiento). Posteriormente se me pedirá que describa mis experiencias únicas durante las situaciones sociales, como mis pensamientos, sentimientos y sensaciones corporales.

Cuando se me planteen preguntas a las que no quiera responder porque me resulten incómodas, puedo simplemente dejarlas en blanco y continuar con la evaluación.

Entiendo que mi participación en este estudio es voluntaria y que puedo retirarme en cualquier momento sin penalización. Participar en esta investigación no tendrá ningún beneficio directo para mí. Sin embargo, los resultados de este estudio pueden ser útiles para la futura comprensión del trastorno de ansiedad social y, por tanto, pueden contribuir a mejorar su tratamiento.

1. Consentimiento informado *

- ◯ He leído este consentimiento y lo entiendo perfectamente. Todas las preguntas relativas a este consentimiento y al estudio han sido respondidas satisfactoriamente y estoy de acuerdo en participar en el estudio.
- ◯ No estoy de acuerdo en participar en este estudio.

Parte I: Escala de Liebowitz para la Ansiedad Social (LSAS)

Esta medida evalúa la forma en que la fobia social desempeña un papel en su vida a través de una variedad de situaciones.

Lea detenidamente cada situación y responda a dos preguntas sobre la misma. La primera pregunta se refiere a lo ansioso o temeroso que se siente en esa situación. La segunda pregunta se refiere a la frecuencia con la que evita la situación.

Si se encuentra con una situación que normalmente no experimenta, imagine "qué pasaría si se enfrentara a esa situación" y, a continuación, puntúe el grado en que temería esta situación hipotética y la frecuencia con la que tendería a evitarla.

Por favor, base sus calificaciones en la forma en que las situaciones le han afectado en la última semana. Rellene la siguiente escala con la respuesta más adecuada que figura a continuación.

Como verá, cada respuesta posible en este cuestionario va seguida de un número entre paréntesis, como por ejemplo (1). Si está interesado en conocer la gravedad de su ansiedad social, anote el número correspondiente después de cada respuesta que seleccione (por ejemplo, 1 - 2 - 0 - 3 - 1 - 3 - 2 - etc.). Al final de esta encuesta, necesitará estos números si quiere interpretar sus puntuaciones (opcional).

2. Llamar por teléfono en presencia de otras personas.

	Nada (0)	Ligero (1)	Moderado (2)	Severo (3)
Miedo o Ansiedad	○	○	○	○

3.

	Nunca: 0% (0)	Ocasionalmente: 1-33% (1)	Con frecuencia: 33-67% (2)	Usualmente: 67-100% (3)
Evitación	○	○	○	○

4. Participar (hablar) en grupos pequeños.

	Nada (0)	Ligero (1)	Moderado (2)	Severo (3)
Miedo o Ansiedad	○	○	○	○

5.

	Nunca: 0% (0)	Ocasionalmente: 1-33% (1)	Con frecuencia: 33-67% (2)	Usualmente: 67-100% (3)
Evitación	○	○	○	○

6. Comer en lugares públicos.

	Nada (0)	Ligero (1)	Moderado (2)	Severo (3)
Miedo o Ansiedad	◯	◯	◯	◯

7.

	Nunca: 0% (0)	Ocasionalmente: 1-33% (1)	Con frecuencia: 33-67% (2)	Usualmente: 67-100% (3)
Evitación	◯	◯	◯	◯

8. Beber con otras personas en lugares públicos.

	Nada (0)	Ligero (1)	Moderado (2)	Severo (3)
Miedo o Ansiedad	◯	◯	◯	◯

9.

	Nunca: 0% (0)	Ocasionalmente: 1-33% (1)	Con frecuencia: 33-67% (2)	Usualmente: 67-100% (3)
Evitación	◯	◯	◯	◯

10. Hablar con personas que tienen autoridad.

	Nada (0)	Ligero (1)	Moderado (2)	Severo (3)
Miedo o Ansiedad	◯	◯	◯	◯

11.

	Nunca: 0% (0)	Ocasionalmente: 1-33% (1)	Con frecuencia: 33-67% (2)	Usualmente: 67-100% (3)
Evitación	◯	◯	◯	◯

12. Actuar, desempeñarse o dar una charla frente a una audiencia.

	Nada (0)	Ligero (1)	Moderado (2)	Severo (3)
Miedo o Ansiedad	◯	◯	◯	◯

13.

	Nunca: 0% (0)	Ocasionalmente: 1-33% (1)	Con frecuencia: 33-67% (2)	Usualmente: 67-100% (3)
Evitación	◯	◯	◯	◯

14. Ir a una fiesta.

	Nada (0)	Ligero (1)	Moderado (2)	Severo (3)
Miedo o Ansiedad	◯	◯	◯	◯

15.

	Nunca: 0% (0)	Ocasionalmente: 1-33% (1)	Con frecuencia: 33-67% (2)	Usualmente: 67-100% (3)
Evitación	◯	◯	◯	◯

16. Trabajar mientras le están observando.

	Nada (0)	Ligero (1)	Moderado (2)	Severo (3)
Miedo o Ansiedad	◯	◯	◯	◯

17.

	Nunca: 0% (0)	Ocasionalmente: 1-33% (1)	Con frecuencia: 33-67% (2)	Usualmente: 67-100% (3)
Evitación	◯	◯	◯	◯

18. Escribir mientras le están observando.

	Nada (0)	Ligero (1)	Moderado (2)	Severo (3)
Miedo o Ansiedad	◯	◯	◯	◯

19.

	Nunca: 0% (0)	Ocasionalmente: 1-33% (1)	Con frecuencia: 33-67% (2)	Usualmente: 67-100% (3)
Evitación	◯	◯	◯	◯

20. Llamar por teléfono a alguien que no conoce muy bien.

	Nada (0)	Ligero (1)	Moderado (2)	Severo (3)
Miedo o Ansiedad	◯	◯	◯	◯

21.

	Nunca: 0% (0)	Ocasionalmente: 1-33% (1)	Con frecuencia: 33-67% (2)	Usualmente: 67-100% (3)
Evitación	◯	◯	◯	◯

22. Hablar con personas que no conoce muy bien.

	Nada (0)	Ligero (1)	Moderado (2)	Severo (3)
Miedo o Ansiedad	◯	◯	◯	◯

23.

	Nunca: 0% (0)	Ocasionalmente: 1-33% (1)	Con frecuencia: 33-67% (2)	Usualmente: 67-100% (3)
Evitación	◯	◯	◯	◯

24. Reunirse con personas desconocidas.

	Nada (0)	Ligero (1)	Moderado (2)	Severo (3)
Miedo o Ansiedad	◯	◯	◯	◯

25.

	Nunca: 0% (0)	Ocasionalmente: 1-33% (1)	Con frecuencia: 33-67% (2)	Usualmente: 67-100% (3)
Evitación	◯	◯	◯	◯

26. Orinar en baños públicos.

	Nada (0)	Ligero (1)	Moderado (2)	Severo (3)
Miedo o Ansiedad	◯	◯	◯	◯

27.

	Nunca: 0% (0)	Ocasionalmente: 1-33% (1)	Con frecuencia: 33-67% (2)	Usualmente: 67-100% (3)
Evitación	◯	◯	◯	◯

28. Entrar a una habitación donde el resto de las personas ya está sentada.

	Nada (0)	Ligero (1)	Moderado (2)	Severo (3)
Miedo o Ansiedad	◯	◯	◯	◯

29.

	Nunca: 0% (0)	Ocasionalmente: 1-33% (1)	Con frecuencia: 33-67% (2)	Usualmente: 67-100% (3)
Evitación	◯	◯	◯	◯

30. Ser el centro de atención.

	Nada (0)	Ligero (1)	Moderado (2)	Severo (3)
Miedo o Ansiedad	◯	◯	◯	◯

31.

	Nunca: 0% (0)	Ocasionalmente: 1-33% (1)	Con frecuencia: 33-67% (2)	Usualmente: 67-100% (3)
Evitación	◯	◯	◯	◯

32. Tomar la palabra en una reunión.

	Nada (0)	Ligero (1)	Moderado (2)	Severo (3)
Miedo o Ansiedad	◯	◯	◯	◯

33.

	Nunca: 0% (0)	Ocasionalmente: 1-33% (1)	Con frecuencia: 33-67% (2)	Usualmente: 67-100% (3)
Evitación	◯	◯	◯	◯

34. Realizar un examen, test o prueba.

	Nada (0)	Ligero (1)	Moderado (2)	Severo (3)
Miedo o Ansiedad	◯	◯	◯	◯

35.

	Nunca: 0% (0)	Ocasionalmente: 1-33% (1)	Con frecuencia: 33-67% (2)	Usualmente: 67-100% (3)
Evitación	◯	◯	◯	◯

36. Expresar desacuerdo o desaprobación a personas que no conoce muy bien.

	Nada (0)	Ligero (1)	Moderado (2)	Severo (3)
Miedo o Ansiedad	◯	◯	◯	◯

37.

	Nunca: 0% (0)	Ocasionalmente: 1-33% (1)	Con frecuencia: 33-67% (2)	Usualmente: 67-100% (3)
Evitación	◯	◯	◯	◯

38. Mirar a los ojos de alguien que no conoce muy bien.

	Nada (0)	Ligero (1)	Moderado (2)	Severo (3)
Miedo o Ansiedad	◯	◯	◯	◯

39.

	Nunca: 0% (0)	Ocasionalmente: 1-33% (1)	Con frecuencia: 33-67% (2)	Usualmente: 67-100% (3)
Evitación	◯	◯	◯	◯

40. Presentar un informe a un grupo.

	Nada (0)	Ligero (1)	Moderado (2)	Severo (3)
Miedo o Ansiedad	◯	◯	◯	◯

41.

	Nunca: 0% (0)	Ocasionalmente: 1-33% (1)	Con frecuencia: 33-67% (2)	Usualmente: 67-100% (3)
Evitación	◯	◯	◯	◯

42. Intentar seducir a alguien que le atrae.

	Nada (0)	Ligero (1)	Moderado (2)	Severo (3)
Miedo o Ansiedad	◯	◯	◯	◯

43.

	Nunca: 0% (0)	Ocasionalmente: 1-33% (1)	Con frecuencia: 33-67% (2)	Usualmente: 67-100% (3)
Evitación	◯	◯	◯	◯

44. Devolver una compra a una tienda.

	Nada (0)	Ligero (1)	Moderado (2)	Severo (3)
Miedo o Ansiedad	◯	◯	◯	◯

45.

	Nunca: 0% (0)	Ocasionalmente: 1-33% (1)	Con frecuencia: 33-67% (2)	Usualmente: 67-100% (3)
Evitación	◯	◯	◯	◯

46. Dar una fiesta.

	Nada (0)	Ligero (1)	Moderado (2)	Severo (3)
Miedo o Ansiedad	◯	◯	◯	◯

47.

	Nunca: 0% (0)	Ocasionalmente: 1-33% (1)	Con frecuencia: 33-67% (2)	Usualmente: 67-100% (3)
Evitación	◯	◯	◯	◯

48. Resistir la presión de un vendedor insistente.

	Nada (0)	Ligero (1)	Moderado (2)	Severo (3)
Miedo o Ansiedad	◯	◯	◯	◯

49.

	Nunca: 0% (0)	Ocasionalmente: 1-33% (1)	Con frecuencia: 33-67% (2)	Usualmente: 67-100% (3)
Evitación	◯	◯	◯	◯

Parte II: Cuestionario de Aceptación Acción II (AAQ-II)

Acaba de completar la parte más extensa de la encuesta. Por favor, continúe, ¡las siguientes partes son mucho más cortas!

Además, a partir de ahora, no es necesario que lleve la cuenta de los números entre paréntesis detrás de cada respuesta (esto sólo se aplicaba al primer cuestionario).

Debajo encontrará una lista de afirmaciones. Por favor, puntúe en qué grado cada afirmación ES VERDAD PARA USTED marcando el número correspondiente. Utilice la siguiente escala para hacer su elección.

1 = Nunca es verdad
2 = Muy raramente es verdad
3 = Raramente es verdad
4 = A veces es verdad
5 = Frecuentemente es verdad
6 = Casi siempre es verdad
7 = Siempre es verdad

50.

	Nunca es verdad (1)	Muy raramente es verdad (2)	Raramente es verdad (3)	A veces es verdad (4)	Frecuentemente es verdad (5)	Casi siempre es verdad (6)	Siempre es verdad (7)
Mis experiencias y recuerdos dolorosos hacen que me sea difícil vivir la vida que querría.	○	○	○	○	○	○	○

51.

	Nunca es verdad (1)	Muy raramente es verdad (2)	Raramente es verdad (3)	A veces es verdad (4)	Frecuentemente es verdad (5)	Casi siempre es verdad (6)	Siempre es verdad (7)
Tengo miedo de mis sentimientos.	○	○	○	○	○	○	○

52.

	Nunca es verdad (1)	Muy raramente es verdad (2)	Raramente es verdad (3)	A veces es verdad (4)	Frecuentemente es verdad (5)	Casi siempre es verdad (6)	Siempre es verdad (7)
Me preocupa no ser capaz de controlar mis preocupaciones y sentimientos.	○	○	○	○	○	○	○

53.

	Nunca es verdad (1)	Muy raramente es verdad (2)	Raramente es verdad (3)	A veces es verdad (4)	Frecuentemente es verdad (5)	Casi siempre es verdad (6)	Siempre es verdad (7)
Mis recuerdos dolorosos me impiden llevar una vida plena.	◯	◯	◯	◯	◯	◯	◯

54.

	Nunca es verdad (1)	Muy raramente es verdad (2)	Raramente es verdad (3)	A veces es verdad (4)	Frecuentemente es verdad (5)	Casi siempre es verdad (6)	Siempre es verdad (7)
Mis emociones interfieren en cómo me gustaría que fuera mi vida.	◯	◯	◯	◯	◯	◯	◯

55.

	Nunca es verdad (1)	Muy raramente es verdad (2)	Raramente es verdad (3)	A veces es verdad (4)	Frecuentemente es verdad (5)	Casi siempre es verdad (6)	Siempre es verdad (7)
Parece que la mayoría de la gente lleva su vida mejor que yo.	◯	◯	◯	◯	◯	◯	◯

56.

	Nunca es verdad (1)	Muy raramente es verdad (2)	Raramente es verdad (3)	A veces es verdad (4)	Frecuentemente es verdad (5)	Casi siempre es verdad (6)	Siempre es verdad (7)
Mis preocupaciones interfieren en el camino de lo que quiero conseguir.	◯	◯	◯	◯	◯	◯	◯

Parte III: Anxiety Sensitivity Index 3 (ASI-III)

Este es el último cuestionario. Cuando haya terminado con esta parte, sólo le esperan unas breves preguntas abiertas y habrá terminado.

De nuevo, no es necesario que lleve la cuenta de sus puntuaciones aquí anotándolas.

Responda seleccionando el número (0, 1, 2, 3, 4) que mejor refleje su experiencia con lo que se indica en cada uno de los enunciados. Si algo de lo que se dice no lo ha sentido o experimentado nunca (p.ej., desmayarse en público), conteste como usted crea que se sentiría si realmente le hubiera ocurrido.

Responda a todos los enunciados teniendo en cuenta la siguiente valoración:

0 = Nada o casi nada
1 = Un poco
2 = Bastante
3 = Mucho
4 = Muchísimo

57.

	Nada o casi nada (0)	Un poco (1)	Bastante (2)	Mucho (3)	Muchísimo (4)
Para mí es importante no dar la impresión de estar nervioso/a	◯	◯	◯	◯	◯

58.

	Nada o casi nada (0)	Un poco (1)	Bastante (2)	Mucho (3)	Muchísimo (4)
Cuando no puedo mantener mi mente concentrada en una tarea, siento la preocupación de que podría estar volviéndome loco/a	◯	◯	◯	◯	◯

59.

	Nada o casi nada (0)	Un poco (1)	Bastante (2)	Mucho (3)	Muchísimo (4)
Me asusto cuando mi corazón late de forma rápida	◯	◯	◯	◯	◯

60.

	Nada o casi nada (0)	Un poco (1)	Bastante (2)	Mucho (3)	Muchísimo (4)
Cuando siento malestar en el estómago, me preocupa estar seriamente enfermo/a	◯	◯	◯	◯	◯

61.

	Nada o casi nada (0)	Un poco (1)	Bastante (2)	Mucho (3)	Muchísimo (4)
Me asusto cuando soy incapaz de mantener mi mente concentrada en una tarea	◯	◯	◯	◯	◯

62.

	Nada o casi nada (0)	Un poco (1)	Bastante (2)	Mucho (3)	Muchísimo (4)
Cuando tiemblo en presencia de otras personas, me da miedo lo que puedan pensar de mí	◯	◯	◯	◯	◯

63.

	Nada o casi nada (0)	Un poco (1)	Bastante (2)	Mucho (3)	Muchísimo (4)
Cuando siento opresión en el pecho, me asusta no poder respirar bien	◯	◯	◯	◯	◯

64.

	Nada o casi nada (0)	Un poco (1)	Bastante (2)	Mucho (3)	Muchísimo (4)
Cuando siento dolor en el pecho, me preocupa que vaya a darme un ataque cardíaco	◯	◯	◯	◯	◯

65.

	Nada o casi nada (0)	Un poco (1)	Bastante (2)	Mucho (3)	Muchísimo (4)
Me preocupa que otras personas noten mi ansiedad	◯	◯	◯	◯	◯

66.

	Nada o casi nada (0)	Un poco (1)	Bastante (2)	Mucho (3)	Muchísimo (4)
Cuando tengo la sensación de que las cosas no son reales, me preocupa que pueda estar mentalmente enfermo/a	◯	◯	◯	◯	◯

67.

	Nada o casi nada (0)	Un poco (1)	Bastante (2)	Mucho (3)	Muchísimo (4)
Tengo miedo a sonrojarme delante de la gente	◯	◯	◯	◯	◯

68.

	Nada o casi nada (0)	Un poco (1)	Bastante (2)	Mucho (3)	Muchísimo (4)
Cuando noto que mi corazón da un salto o late de forma irregular, me preocupa que algo grave me esté ocurriendo	◯	◯	◯	◯	◯

69.

	Nada o casi nada (0)	Un poco (1)	Bastante (2)	Mucho (3)	Muchísimo (4)
Cuando comienzo a sudar en una situación social, me da miedo que la gente piense negativamente de mí	◯	◯	◯	◯	◯

70.

	Nada o casi nada (0)	Un poco (1)	Bastante (2)	Mucho (3)	Muchísimo (4)
Cuando mis pensamientos parecen acelerarse, me preocupa que pueda volverme loco/a	◯	◯	◯	◯	◯

71.

	Nada o casi nada (0)	Un poco (1)	Bastante (2)	Mucho (3)	Muchísimo (4)
Cuando siento opresión en la garganta, me preocupa que pueda atragantarme y morir	◯	◯	◯	◯	◯

72.

	Nada o casi nada (0)	Un poco (1)	Bastante (2)	Mucho (3)	Muchísimo (4)
Cuando me resulta difícil pensar con claridad, me preocupa que me esté ocurriendo algo grave	○	○	○	○	○

73.

	Nada o casi nada (0)	Un poco (1)	Bastante (2)	Mucho (3)	Muchísimo (4)
Pienso que me resultaría horrible si me desmayase en público	○	○	○	○	○

74.

	Nada o casi nada (0)	Un poco (1)	Bastante (2)	Mucho (3)	Muchísimo (4)
Cuando mi mente se queda en blanco, me preocupa que me esté ocurriendo algo terriblemente malo	○	○	○	○	○

Parte IV: Preguntas abiertas

¡Ya casi ha terminado! Esta sección evalúa sus experiencias con pensamientos, sentimientos y sensaciones físicas incómodas e intrusivas.

No hay una forma correcta o incorrecta de comprender y responder a estas preguntas. La idea aquí es que elabore libremente sobre sus experiencias, sin pensar demasiado en si lo está haciendo bien o mal.

Cuanto más detalladas sean sus respuestas, más valiosas serán para nuestro análisis. Gracias por tomarse el tiempo.

75. Cuando se encuentra en una situación social que percibe como estresante, ¿suele experimentar algo de lo siguiente?

    -Pensamientos indeseados o incómodos que parecen surgir de la nada.

    -Sentimientos o emociones no deseadas o incómodas.

    -Reacciones corporales no deseadas o incómodas o manifestaciones físicas de ansiedad.

    En caso afirmativo, nombre y describa estos fenómenos.

76. ¿Cómo le afecta cuando nota que surgen pensamientos, sentimientos o reacciones corporales no deseados o incómodos durante una situación social?

77. ¿Hay algún tipo específico de situación social en la que experimente pensamientos, sentimientos o reacciones corporales no deseados o incómodos como particularmente molestos?

78. Cuando nota que surgen pensamientos, sentimientos o reacciones corporales no deseados o incómodos durante una situación social, ¿cómo suele reaccionar?

79. ¿Cómo afecta a su ansiedad o nerviosismo la forma en que responde a pensamientos, sentimientos o reacciones corporales no deseados o incómodos durante las situaciones sociales?

80. Seleccione la opción que mejor describa su caso.

- Experimento miedo o ansiedad en prácticamente todas las situaciones sociales.
- Experimento miedo o ansiedad en un número considerable de situaciones sociales.
- Experimento miedo o ansiedad en una o muy pocas situaciones sociales.
- Nada de lo anterior.

81. Seleccione la opción que mejor describa su caso.

- En situaciones sociales, me preocupa principalmente comportarme de forma inepta o tonta (como decir algo fuera de lugar o algo que pueda ser percibido como "estúpido", etc.).
- En situaciones sociales, me preocupa sobre todo mostrar signos observables de ansiedad (como sudoración excesiva, rubor, voz temblorosa, etc.).
- En situaciones sociales, me preocupa sobre todo la posibilidad de ofender a los demás (como decir algo que pueda herir sus sentimientos, etc.).
- Nada de lo anterior.

82. Por favor, indique su nacionalidad.

83. Por favor, indique su edad.

This content is neither created nor endorsed by Microsoft. The data you submit will be sent to the form owner.

Microsoft Forms

Questionário de Pesquisa de Ansiedade Social (Português)

Este questionário foi elaborado para explorar as experiências de pessoas que sofrem de ansiedade social. Ele consiste de quatro partes e leva cerca de 15 minutos para ser concluído.

A última parte do questionário é composta de várias perguntas abertas que se destinam a permitir que você desenvolva livremente as suas experiências. Quanto mais detalhadas suas respostas estiverem nesta parte da pesquisa, mais valiosas elas serão para nossa análise.

Suas respostas nesta pesquisa serão anônimas. Você pode ter certeza de que nenhuma informação pessoal será associada com os resultados da pesquisa.

Se você estiver interessado em ter uma idéia da gravidade de sua ansiedade social, você pode manter um registro de suas pontuações escrevendo-as em um pedaço de papel ou em seu telefone. Você será lembrado quando relevante.

Obrigado por dedicar seu tempo para participar deste estudo.

* Required

Participação no estudo: Consentimento informado

Fui oferecido para participar desta pesquisa, que será conduzida sob a direção de Martin Stork, estudante de psicologia, e Mariantonia Lemos Hoyos, psicóloga e professora, na Universidade EAFIT, Colômbia.

O presente estudo visa examinar até que ponto indivíduos com ansiedade social controlam eventos internos tais como pensamentos, sentimentos e sensações físicas durante situações sociais. Também visa identificar as crenças e atitudes de indivíduos com ansiedade social em relação às suas tentativas de monitorar e controlar suas experiências internas em situações sociais, bem como explorar as possíveis estratégias que eles empregam para tentar fazer isso.

Este estudo é parte do trabalho de graduação do estudante de psicologia Martin Stork.

Fui informado de que no decorrer deste estudo me serão apresentados questionários curtos nos quais serão avaliados diferentes aspectos psicológicos (ansiedade, percepção de sentimentos desconfortáveis, estratégias de enfrentamento). Posteriormente, serei solicitado a descrever minhas experiências únicas durante situações sociais, tais como meus pensamentos, sentimentos e sensações corporais.

Quando me fazem perguntas que não quero responder porque são desconfortáveis, posso simplesmente deixá-las em branco e continuar com a avaliação.

Eu entendo que minha participação neste estudo é voluntária e que eu posso me retirar a qualquer momento sem penalidades. A participação nesta pesquisa não terá nenhum benefício direto para mim. No entanto, os resultados deste estudo podem ser úteis para a compreensão futura do transtorno de ansiedade social e, portanto, podem contribuir para melhorar seu tratamento.

1. Consentimento informado *

- ◯ Eu li este formulário de consentimento e o entendi totalmente. Todas as perguntas relativas a este consentimento e ao estudo foram respondidas satisfatoriamente e eu concordo em participar do estudo.
- ◯ Eu não concordo em participar deste estudo.

Parte I: Escala de Ansiedade Social de Liebowitz (LSAS-SR)

Esta medida avalia a forma como a fobia social desempenha um papel na sua vida através de uma variedade de situações.

Leia cada situação cuidadosamente e responda duas perguntas sobre essa situação. A primeira pergunta lhe questiona quão ansioso ou temeroso você se sente na situação. A segunda pergunta se refere à freqüência com a qual você evita a situação.

Se você se deparar com uma situação que você normalmente não experimenta, imagine "e se você fosse confrontado com aquela situação" e então, avalie o grau em que você temeria esta situação hipotética e com que freqüência você tenderia a evitá-la.

Por favor, baseie suas avaliações na forma como as situações o afetaram na última semana. Preencha a seguinte escala com a resposta mais adequada fornecida abaixo.

Como você verá, cada resposta possível neste questionário é seguida por um número entre parênteses, tal como (1). Se você estiver interessado em saber a gravidade de sua ansiedade social, anote o número correspondente após cada resposta que você selecionar (por exemplo, 1 - 2 - 0 - 3 - 1 - 3 - 2 - etc.). Ao final desta pesquisa, você precisará destes números se você quiser interpretar suas pontuações (opcional).

2. Telefonar em público.

	Nenhum (0)	Leve (1)	Moderado (2)	Intenso (3)
Medo ou Ansiedade	○	○	○	○

3.

	Nunca: 0% (0)	Ocasionalmente: 1-33% (1)	Freqüentemente: 33-67% (2)	Geralmente: 67-100% (3)
Evitação	○	○	○	○

4. Participar de pequenos grupos.

	Nenhum (0)	Leve (1)	Moderado (2)	Intenso (3)
Medo ou Ansiedade	○	○	○	○

5.

	Nunca: 0% (0)	Ocasionalmente: 1-33% (1)	Freqüentemente: 33-67% (2)	Geralmente: 67-100% (3)
Evitação	○	○	○	○

6. Comer em locais públicos.

	Nenhum (0)	Leve (1)	Moderado (2)	Intenso (3)
Medo ou Ansiedade	◯	◯	◯	◯

7.

	Nunca: 0% (0)	Ocasionalmente: 1-33% (1)	Freqüentemente: 33-67% (2)	Geralmente: 67-100% (3)
Evitação	◯	◯	◯	◯

8. Beber com outros em locais públicos.

	Nenhum (0)	Leve (1)	Moderado (2)	Intenso (3)
Medo ou Ansiedade	◯	◯	◯	◯

9.

	Nunca: 0% (0)	Ocasionalmente: 1-33% (1)	Freqüentemente: 33-67% (2)	Geralmente: 67-100% (3)
Evitação	◯	◯	◯	◯

10. Falar com pessoas em posição de autoridade.

	Nenhum (0)	Leve (1)	Moderado (2)	Intenso (3)
Medo ou Ansiedade	◯	◯	◯	◯

11.

	Nunca: 0% (0)	Ocasionalmente: 1-33% (1)	Freqüentemente: 33-67% (2)	Geralmente: 67-100% (3)
Evitação	◯	◯	◯	◯

12. Agir, realizar ou falar em frente a uma audiência.

	Nenhum (0)	Leve (1)	Moderado (2)	Intenso (3)
Medo ou Ansiedade	◯	◯	◯	◯

13.

	Nunca: 0% (0)	Ocasionalmente: 1-33% (1)	Freqüentemente: 33-67% (2)	Geralmente: 67-100% (3)
Evitação	◯	◯	◯	◯

14. Ir a uma festa.

	Nenhum (0)	Leve (1)	Moderado (2)	Intenso (3)
Medo ou Ansiedade	◯	◯	◯	◯

15.

	Nunca: 0% (0)	Ocasionalmente: 1-33% (1)	Freqüentemente: 33-67% (2)	Geralmente: 67-100% (3)
Evitação	◯	◯	◯	◯

16. Trabalhar sendo observado.

	Nenhum (0)	Leve (1)	Moderado (2)	Intenso (3)
Medo ou Ansiedade	◯	◯	◯	◯

17.

	Nunca: 0% (0)	Ocasionalm ente: 1-33% (1)	Freqüente mente: 33-67% (2)	Geralmente : 67-100% (3)
Evitação	◯	◯	◯	◯

18. Escrever sendo observado.

	Nenhum (0)	Leve (1)	Moderado (2)	Intenso (3)
Medo ou Ansiedade	◯	◯	◯	◯

19.

	Nunca: 0% (0)	Ocasionalm ente: 1-33% (1)	Freqüente mente: 33-67% (2)	Geralmente : 67-100% (3)
Evitação	◯	◯	◯	◯

20. Chamar alguém que você não conhece muito bem.

	Nenhum (0)	Leve (1)	Moderado (2)	Intenso (3)
Medo ou Ansiedade	◯	◯	◯	◯

21.

	Nunca: 0% (0)	Ocasionalmente: 1-33% (1)	Freqüentemente: 33-67% (2)	Geralmente: 67-100% (3)
Evitação	◯	◯	◯	◯

22. Falar com pessoas que você não conhece muito bem.

	Nenhum (0)	Leve (1)	Moderado (2)	Intenso (3)
Medo ou Ansiedade	◯	◯	◯	◯

23.

	Nunca: 0% (0)	Ocasionalmente: 1-33% (1)	Freqüentemente: 33-67% (2)	Geralmente: 67-100% (3)
Evitação	◯	◯	◯	◯

24. Encontrar com estranhos.

	Nenhum (0)	Leve (1)	Moderado (2)	Intenso (3)
Medo ou Ansiedade	◯	◯	◯	◯

25.

	Nunca: 0% (0)	Ocasionalmente: 1-33% (1)	Freqüentemente: 33-67% (2)	Geralmente: 67-100% (3)
Evitação	◯	◯	◯	◯

26. Urinar em banheiro público.

	Nenhum (0)	Leve (1)	Moderado (2)	Intenso (3)
Medo ou Ansiedade	◯	◯	◯	◯

27.

	Nunca: 0% (0)	Ocasionalmente: 1-33% (1)	Freqüentemente: 33-67% (2)	Geralmente: 67-100% (3)
Evitação	◯	◯	◯	◯

28. Entrar em uma sala onde outros já estão sentados.

	Nenhum (0)	Leve (1)	Moderado (2)	Intenso (3)
Medo ou Ansiedade	◯	◯	◯	◯

29.

	Nunca: 0% (0)	Ocasionalmente: 1-33% (1)	Freqüentemente: 33-67% (2)	Geralmente: 67-100% (3)
Evitação	◯	◯	◯	◯

30. Ser o centro das atenções.

	Nenhum (0)	Leve (1)	Moderado (2)	Intenso (3)
Medo ou Ansiedade	◯	◯	◯	◯

31.

	Nunca: 0% (0)	Ocasionalmente: 1-33% (1)	Freqüentemente: 33-67% (2)	Geralmente: 67-100% (3)
Evitação	◯	◯	◯	◯

32. Falar em uma reunião.

	Nenhum (0)	Leve (1)	Moderado (2)	Intenso (3)
Medo ou Ansiedade	◯	◯	◯	◯

33.

	Nunca: 0% (0)	Ocasionalmente: 1-33% (1)	Freqüentemente: 33-67% (2)	Geralmente: 67-100% (3)
Evitação	◯	◯	◯	◯

34. Fazer uma prova.

	Nenhum (0)	Leve (1)	Moderado (2)	Intenso (3)
Medo ou Ansiedade	◯	◯	◯	◯

35.

	Nunca: 0% (0)	Ocasionalmente: 1-33% (1)	Freqüentemente: 33-67% (2)	Geralmente: 67-100% (3)
Evitação	◯	◯	◯	◯

36. Expressar uma discordância ou desaprovação para pessoas que você não conhece bem.

	Nenhum (0)	Leve (1)	Moderado (2)	Intenso (3)
Medo ou Ansiedade	◯	◯	◯	◯

37.

	Nunca: 0% (0)	Ocasionalmente: 1-33% (1)	Freqüentemente: 33-67% (2)	Geralmente: 67-100% (3)
Evitação	◯	◯	◯	◯

38. Olhar nos olhos de pessoas que você não conheça bem.

	Nenhum (0)	Leve (1)	Moderado (2)	Intenso (3)
Medo ou Ansiedade	◯	◯	◯	◯

39.

	Nunca: 0% (0)	Ocasionalmente: 1-33% (1)	Freqüentemente: 33-67% (2)	Geralmente: 67-100% (3)
Evitação	◯	◯	◯	◯

40. Relatar algo para um grupo.

	Nenhum (0)	Leve (1)	Moderado (2)	Intenso (3)
Medo ou Ansiedade	◯	◯	◯	◯

41.

	Nunca: 0% (0)	Ocasionalmente: 1-33% (1)	Freqüentemente: 33-67% (2)	Geralmente: 67-100% (3)
Evitação	◯	◯	◯	◯

42. Tentar paquerar alguém.

	Nenhum (0)	Leve (1)	Moderado (2)	Intenso (3)
Medo ou Ansiedade	◯	◯	◯	◯

43.

	Nunca: 0% (0)	Ocasionalmente: 1-33% (1)	Freqüentemente: 33-67% (2)	Geralmente: 67-100% (3)
Evitação	◯	◯	◯	◯

44. Devolver mercadorias para uma loja.

	Nenhum (0)	Leve (1)	Moderado (2)	Intenso (3)
Medo ou Ansiedade	◯	◯	◯	◯

45.

	Nunca: 0% (0)	Ocasionalmente: 1-33% (1)	Freqüentemente: 33-67% (2)	Geralmente: 67-100% (3)
Evitação	◯	◯	◯	◯

46. Dar uma festa.

	Nenhum (0)	Leve (1)	Moderado (2)	Intenso (3)
Medo ou Ansiedade	◯	◯	◯	◯

47.

	Nunca: 0% (0)	Ocasionalmente: 1-33% (1)	Freqüentemente: 33-67% (2)	Geralmente: 67-100% (3)
Evitação	◯	◯	◯	◯

48. Resistir as pressões de um vendedor.

	Nenhum (0)	Leve (1)	Moderado (2)	Intenso (3)
Medo ou Ansiedade	◯	◯	◯	◯

49.

	Nunca: 0% (0)	Ocasionalmente: 1-33% (1)	Freqüentemente: 33-67% (2)	Geralmente: 67-100% (3)
Evitação	◯	◯	◯	◯

Parte II: Questionário de Aceitação e Ação (AAQ II)

Você acabou de completar a parte mais extensa da pesquisa. Por favor, continue, as próximas partes são muito mais curtas!

Além disso, de agora em diante, você não precisa registrar os números entre parênteses atrás de cada resposta (isso só se aplica ao primeiro questionário).

A seguir, você encontrará uma lista de afirmações. Por favor, avalie quanto cada afirmação é verdadeira para você e selecione o número correspondente. Use a escala abaixo para fazer sua escolha.

1 = Nunca
2 = Muito raramente
3 = Raramente
4 = Algumas vezes
5 = Frequentemente
6 = Quase sempre
7 = Sempre

50.

	Nunca (1)	Muito raramente (2)	Raramente (3)	Algumas vezes (4)	Frequente mente (5)	Quase sempre (6)	Sempre (7)
Minhas experiências e lembranças dolorosas dificultam que eu viva a vida que eu gostaria.	○	○	○	○	○	○	○

51.

	Nunca (1)	Muito raramente (2)	Raramente (3)	Algumas vezes (4)	Frequente mente (5)	Quase sempre (6)	Sempre (7)
Tenho medo dos meus sentimentos.	○	○	○	○	○	○	○

52.

	Nunca (1)	Muito raramente (2)	Raramente (3)	Algumas vezes (4)	Frequente mente (5)	Quase sempre (6)	Sempre (7)
Eu me preocupo em não conseguir controlar minhas preocupações e sentimentos.	○	○	○	○	○	○	○

53.

	Nunca (1)	Muito raramente (2)	Raramente (3)	Algumas vezes (4)	Frequente mente (5)	Quase sempre (6)	Sempre (7)
Minhas lembranças dolorosas me impedem de ter uma vida plena.	◯	◯	◯	◯	◯	◯	◯

54.

	Nunca (1)	Muito raramente (2)	Raramente (3)	Algumas vezes (4)	Frequente mente (5)	Quase sempre (6)	Sempre (7)
Emoções causam problemas na minha vida.	◯	◯	◯	◯	◯	◯	◯

55.

	Nunca (1)	Muito raramente (2)	Raramente (3)	Algumas vezes (4)	Frequente mente (5)	Quase sempre (6)	Sempre (7)
Parece que a maioria das pessoas lida com suas vidas melhor do que eu.	◯	◯	◯	◯	◯	◯	◯

56.

	Nunca (1)	Muito raramente (2)	Raramente (3)	Algumas vezes (4)	Frequente mente (5)	Quase sempre (6)	Sempre (7)
Preocupações atrapalham o meu sucesso.	◯	◯	◯	◯	◯	◯	◯

Parte III: Índice de Sensibilidade à Ansiedade 3 (ASI-III)

Este é o último questionário. Quando você terminar esta parte, há apenas algumas breves perguntas abertas esperando por você e você está pronto!

Mais uma vez, você não precisa acompanhar suas pontuações aqui, anotando-as.

Assinale o número que melhor corresponde ao quanto concorda com cada afirmação.

Se alguma afirmação disser respeito a algo que nunca vivenciou (por ex.: desmaiar em público), responda com base no que acha que sentiria se isso tivesse acontecido. Caso contrário, responda a cada afirmação tendo por base a sua experiência pessoal.

Tenha o cuidado de assinalar somente um número para cada afirmação e, por favor, responda a todas as questões.

0 = Concordo muito pouco
1 = Concordo pouco
2 = Concordo moderadamente
3 = Concordo muito
4 = Concordo muitíssimo

57.

	Concordo muito pouco (0)	Concordo pouco (1)	Concordo moderada mente (2)	Concordo muito (3)	Concordo muitíssimo (4)
É importante para mim não parecer nervoso/a.	○	○	○	○	○

58.

	Concordo muito pouco (0)	Concordo pouco (1)	Concordo moderada mente (2)	Concordo muito (3)	Concordo muitíssimo (4)
Quando não me consigo concentrar numa tarefa, preocupo-me se posso estar a enlouquecer.	○	○	○	○	○

59.

	Concordo muito pouco (0)	Concordo pouco (1)	Concordo moderada mente (2)	Concordo muito (3)	Concordo muitíssimo (4)
Fico assustado/a quando o meu coração bate rapidamente.	○	○	○	○	○

60.

	Concordo muito pouco (0)	Concordo pouco (1)	Concordo moderadamente (2)	Concordo muito (3)	Concordo muitíssimo (4)
Quando tenho problemas no estômago, preocupo-me se posso estar gravemente doente.	◯	◯	◯	◯	◯

61.

	Concordo muito pouco (0)	Concordo pouco (1)	Concordo moderadamente (2)	Concordo muito (3)	Concordo muitíssimo (4)
Fico assustado/a quando não sou capaz de me concentrar numa tarefa.	◯	◯	◯	◯	◯

62.

	Concordo muito pouco (0)	Concordo pouco (1)	Concordo moderadamente (2)	Concordo muito (3)	Concordo muitíssimo (4)
Quando tremo à frente de outras pessoas, preocupo-me sobre o que possam pensar de mim.	◯	◯	◯	◯	◯

63.

	Concordo muito pouco (0)	Concordo pouco (1)	Concordo moderadamente (2)	Concordo muito (3)	Concordo muitíssimo (4)
Quando sinto um aperto no peito, assusta-me não conseguir respirar bem.	◯	◯	◯	◯	◯

64.

	Concordo muito pouco (0)	Concordo pouco (1)	Concordo moderadamente (2)	Concordo muito (3)	Concordo muitíssimo (4)
Quando sinto uma dor no peito, preocupo-me se irei ter um enfarte.	○	○	○	○	○

65.

	Concordo muito pouco (0)	Concordo pouco (1)	Concordo moderadamente (2)	Concordo muito (3)	Concordo muitíssimo (4)
Preocupo-me que outras pessoas se apercebam da minha ansiedade.	○	○	○	○	○

66.

	Concordo muito pouco (0)	Concordo pouco (1)	Concordo moderadamente (2)	Concordo muito (3)	Concordo muitíssimo (4)
Quando me sinto ”aluado/a” ou desorientado/a, tenho medo de poder estar com alguma doença mental.	○	○	○	○	○

67.

	Concordo muito pouco (0)	Concordo pouco (1)	Concordo moderadamente (2)	Concordo muito (3)	Concordo muitíssimo (4)
Tenho receio de corar à frente de outras pessoas.	○	○	○	○	○

68.

	Concordo muito pouco (0)	Concordo pouco (1)	Concordo moderadamente (2)	Concordo muito (3)	Concordo muitíssimo (4)
Quando me apercebo de que estou com batimentos cardíacos irregulares, preocupo-me se tenho algo grave.	◯	◯	◯	◯	◯

69.

	Concordo muito pouco (0)	Concordo pouco (1)	Concordo moderadamente (2)	Concordo muito (3)	Concordo muitíssimo (4)
Quando começo a transpirar ao pé de outras pessoas, receio que pensem mal de mim.	◯	◯	◯	◯	◯

70.

	Concordo muito pouco (0)	Concordo pouco (1)	Concordo moderadamente (2)	Concordo muito (3)	Concordo muitíssimo (4)
Quando os meus pensamentos parecem acelerar, preocupo-me se estarei a enlouquecer.	◯	◯	◯	◯	◯

71.

	Concordo muito pouco (0)	Concordo pouco (1)	Concordo moderadamente (2)	Concordo muito (3)	Concordo muitíssimo (4)
Quando sinto a garganta apertada, preocupo-me que possa morrer sufocado/a.	◯	◯	◯	◯	◯

72.

	Concordo muito pouco (0)	Concordo pouco (1)	Concordo moderada mente (2)	Concordo muito (3)	Concordo muitíssimo (4)
Quando tenho dificuldade em pensar com clareza, preocupo-me se terei algum problema.	◯	◯	◯	◯	◯

73.

	Concordo muito pouco (0)	Concordo pouco (1)	Concordo moderada mente (2)	Concordo muito (3)	Concordo muitíssimo (4)
Penso que seria horrível para mim se desmaiasse em público.	◯	◯	◯	◯	◯

74.

	Concordo muito pouco (0)	Concordo pouco (1)	Concordo moderada mente (2)	Concordo muito (3)	Concordo muitíssimo (4)
Quando tenho uma "branca", preocupo-me se tenho algo terrivelmente grave.	◯	◯	◯	◯	◯

Parte IV: Perguntas abertas

Está quase terminando! Esta seção avalia suas experiências com pensamentos desconfortáveis e intrusivos, sentimentos e sensações físicas.

Não existe uma maneira certa ou errada de entender e responder a estas perguntas. A idéia aqui é que você desenvolva livremente suas experiências, sem pensar muito se você está fazendo certo ou errado.

Quanto mais detalhadas forem suas respostas, mais valiosas elas serão para nossa análise. Obrigado por dedicar seu tempo.

75. Quando você está em uma situação social que você percebe como estressante, você freqüentemente experimenta algo do seguinte?

    -Pensamentos indesejados ou desconfortáveis que parecem surgir do nada.

    -Sentimentos ou emoções indesejados ou desconfortáveis.

    -Reações corporais indesejadas ou desconfortáveis ou manifestações físicas de ansiedade.

    Se sim, nomeie e descreva esses fenômenos.

76. Como o afeta quando você percebe que pensamentos, sentimentos ou reações corporais indesejados ou desconfortáveis surgem durante uma situação social?

77. Existe um tipo específico de situação social na qual você experimenta pensamentos, sentimentos ou reações corporais indesejados ou desconfortáveis como particularmente perturbadores?

78. Quando você percebe que pensamentos, sentimentos ou reações corporais indesejados ou desconfortáveis surgem durante uma situação social, como você geralmente reage?

79. Como a forma como você responde aos pensamentos, sentimentos ou reações corporais indesejados ou desconfortáveis durante situações sociais afeta sua ansiedade ou nervosismo?

80. Selecione a opção que melhor descreve o seu caso.

- ◯ Eu sinto medo ou ansiedade em praticamente todas as situações sociais.
- ◯ Eu sinto medo ou ansiedade em um número considerável de situações sociais.
- ◯ Eu sinto medo ou ansiedade em uma ou muito poucas situações sociais.
- ◯ Nenhum dos acima mencionados.

81. Selecione a opção que melhor descreve o seu caso.

- ◯ Em situações sociais, eu estou principalmente preocupado em me comportar de forma inepta ou tola (como dizer algo fora do lugar ou algo que poderia ser percebido como "estúpido", etc.).
- ◯ Em situações sociais, eu estou particularmente preocupado em mostrar sinais observáveis de ansiedade (como suor excessivo, rubor, voz trêmula, etc.).
- ◯ Em situações sociais, eu estou mais preocupado com a possibilidade de ofender os outros (como dizer algo que possa ferir seus sentimentos, etc.).
- ◯ Nenhum dos acima mencionados.

82. Por favor, indique sua nacionalidade.

83. Por favor, indique a sua idade.

This content is neither created nor endorsed by Microsoft. The data you submit will be sent to the form owner.

Microsoft Forms

Questionnaire de recherche sur l'anxiété sociale (français)

Ce questionnaire est conçu pour explorer les expériences des personnes qui souffrent d'anxiété sociale. Il se compose de quatre parties et prend environ 15 minutes à remplir.

La dernière partie du questionnaire comporte plusieurs questions ouvertes destinées à vous permettre de décrire librement vos expériences. Plus vos réponses dans cette partie du questionnaire seront détaillées, plus elles auront de valeur pour notre analyse.

Vos réponses à cette enquête seront anonymes. Soyez assuré qu'aucune information personnelle ne sera associée aux résultats de la recherche.

Si vous souhaitez avoir une idée de la gravité de votre anxiété sociale, vous pouvez suivre vos scores en les notant sur une feuille de papier ou sur votre téléphone. Vous recevrez un rappel lorsque cela sera pertinent.

Merci de prendre le temps de participer à cette étude.

* Required

Participation à l'étude : Consentement éclairé

On m'a proposé de participer à cette recherche, qui sera menée sous la direction de Martin Stork, étudiant en psychologie, et de Mariantonia Lemos Hoyos, psychologue et professeure, à l'Universidad EAFIT, en Colombie.

La présente étude vise à examiner dans quelle mesure les personnes souffrant d'anxiété sociale contrôlent les événements internes tels que les pensées, les sentiments et les sensations physiques pendant les situations sociales. Elle vise également à identifier les croyances et les attitudes des individus souffrant d'anxiété sociale concernant leurs tentatives de surveiller et de contrôler leurs expériences internes dans les situations sociales, ainsi qu'à explorer toute stratégie potentielle qu'ils pourraient employer pour tenter de le faire.

Cette étude fait partie de la dissertation de l'étudiant en psychologie Martin Stork.

J'ai été informé qu'au cours de la présente étude, on me présentera de brefs questionnaires évaluant différents aspects psychologiques (anxiété, perceptions de sentiments inconfortables, stratégies d'adaptation). On me demandera ensuite de décrire mes expériences particulières lors de situations sociales, telles que mes pensées, mes sentiments et mes sensations corporelles.

Lorsque je suis confronté à des questions auxquelles je ne veux pas répondre parce qu'elles me mettent mal à l'aise, je peux simplement les laisser en blanc et poursuivre l'évaluation.

Je comprends que ma participation à cette étude est volontaire et que je peux me retirer à tout moment sans pénalité. La participation à cette recherche n'aura aucun avantage direct pour moi. Cependant, les résultats de cette étude peuvent être utiles pour la compréhension future du trouble d'anxiété sociale et, par conséquent, peuvent contribuer à améliorer son traitement.

1. Consentement éclairé *

- ◯ J'ai lu ce formulaire de consentement et je le comprends parfaitement. Toutes les questions concernant ce consentement et l'étude ont reçu une réponse satisfaisante et j'accepte de participer à l'étude.
- ◯ Je n'accepte pas de participer à cette étude.

Partie I : Échelle d'anxiété sociale de Liebowitz (LSAS-SR)

Ce questionnaire évalue la façon dont la phobie sociale joue un rôle dans votre vie à travers une variété de situations.

Lisez attentivement chaque situation et répondez à deux questions sur cette situation. La première question demande à quel point vous vous sentez anxieux ou craintif dans cette situation. La deuxième question demande à quelle fréquence vous évitez la situation.

Si vous rencontrez une situation que vous ne vivez pas habituellement, imaginez "que se passerait-il si vous étiez confronté à cette situation", puis évaluez dans quelle mesure vous craindriez cette situation hypothétique et à quelle fréquence vous auriez tendance à l'éviter.

Veuillez baser vos évaluations sur la façon dont les situations vous ont affecté au cours de la semaine dernière. Remplissez l'échelle suivante avec la réponse la plus appropriée fournie ci-dessous.

Comme vous le verrez, chaque réponse possible dans ce questionnaire est suivie d'un chiffre entre parenthèses, comme par exemple (1). Si vous souhaitez connaître la gravité de votre anxiété sociale, inscrivez le chiffre correspondant à la suite de chaque réponse que vous sélectionnez (par exemple, 1 - 2 - 0 - 3 - 1 - 3 - 2 - etc.). À la fin de cette enquête, vous aurez besoin de ces chiffres si vous voulez interpréter vos scores (facultatif).

2. Téléphoner en public

	Aucune (0)	Légère (1)	Moyenne (2)	Sévère (3)
Anxiété	○	○	○	○

3.

	Jamais: 0% (0)	Occasionnel: 1-33% (1)	Fréquent: 33-67% (2)	Habituel: 67-100% (3)
Evitement	○	○	○	○

4. Participer à des activités en petits groupes

	Aucune (0)	Légère (1)	Moyenne (2)	Sévère (3)
Anxiété	○	○	○	○

5.

	Jamais: 0% (0)	Occasionnel: 1-33% (1)	Fréquent: 33-67% (2)	Habituel: 67-100% (3)
Evitement	○	○	○	○

6. Manger dans des lieux publics

	Aucune (0)	Légère (1)	Moyenne (2)	Sévère (3)
Anxiété	○	○	○	○

7.

	Jamais: 0% (0)	Occasionnel: 1-33% (1)	Fréquent: 33-67% (2)	Habituel: 67-100% (3)
Evitement	○	○	○	○

8. Boire avec d'autres personnes dans des lieux publics

	Aucune (0)	Légère (1)	Moyenne (2)	Sévère (3)
Anxiété	○	○	○	○

9.

	Jamais: 0% (0)	Occasionnel: 1-33% (1)	Fréquent: 33-67% (2)	Habituel: 67-100% (3)
Evitement	○	○	○	○

10. Parler à quelqu'un en position d'autorité

	Aucune (0)	Légère (1)	Moyenne (2)	Sévère (3)
Anxiété	○	○	○	○

11.

	Jamais: 0% (0)	Occasionnel: 1-33% (1)	Fréquent: 33-67% (2)	Habituel: 67-100% (3)
Evitement	○	○	○	○

12. Jouer la comédie, effectuer une performance, ou parler devant un auditoire

	Aucune (0)	Légère (1)	Moyenne (2)	Sévère (3)
Anxiété	◯	◯	◯	◯

13.

	Jamais: 0% (0)	Occasionnel: 1-33% (1)	Fréquent: 33-67% (2)	Habituel: 67-100% (3)
Evitement	◯	◯	◯	◯

14. Aller à une soirée

	Aucune (0)	Légère (1)	Moyenne (2)	Sévère (3)
Anxiété	◯	◯	◯	◯

15.

	Jamais: 0% (0)	Occasionnel: 1-33% (1)	Fréquent: 33-67% (2)	Habituel: 67-100% (3)
Evitement	◯	◯	◯	◯

16. Travailler en étant observé(e)

	Aucune (0)	Légère (1)	Moyenne (2)	Sévère (3)
Anxiété	◯	◯	◯	◯

17.

	Jamais: 0% (0)	Occasionnel: 1-33% (1)	Fréquent: 33-67% (2)	Habituel: 67-100% (3)
Evitement	◯	◯	◯	◯

18. Ecrire en étant observé(e)

	Aucune (0)	Légère (1)	Moyenne (2)	Sévère (3)
Anxiété	◯	◯	◯	◯

19.

	Jamais: 0% (0)	Occasionnel: 1-33% (1)	Fréquent: 33-67% (2)	Habituel: 67-100% (3)
Evitement	◯	◯	◯	◯

20. Téléphoner à une personne que vous ne connaissez pas très bien

	Aucune (0)	Légère (1)	Moyenne (2)	Sévère (3)
Anxiété	◯	◯	◯	◯

21.

	Jamais: 0% (0)	Occasionnel: 1-33% (1)	Fréquent: 33-67% (2)	Habituel: 67-100% (3)
Evitement	◯	◯	◯	◯

22. Parler à une personne que vous ne connaissez pas très bien

	Aucune (0)	Légère (1)	Moyenne (2)	Sévère (3)
Anxiété	◯	◯	◯	◯

23.

	Jamais: 0% (0)	Occasionnel: 1-33% (1)	Fréquent: 33-67% (2)	Habituel: 67-100% (3)
Evitement	◯	◯	◯	◯

24. Rencontrer des inconnu(e)s

	Aucune (0)	Légère (1)	Moyenne (2)	Sévère (3)
Anxiété	◯	◯	◯	◯

25.

	Jamais: 0% (0)	Occasionnel: 1-33% (1)	Fréquent: 33-67% (2)	Habituel: 67-100% (3)
Evitement	◯	◯	◯	◯

26. Uriner dans les toilettes publiques

	Aucune (0)	Légère (1)	Moyenne (2)	Sévère (3)
Anxiété	◯	◯	◯	◯

27.

	Jamais: 0% (0)	Occasionnel: 1-33% (1)	Fréquent: 33-67% (2)	Habituel: 67-100% (3)
Evitement	◯	◯	◯	◯

28. Entrer dans une pièce où les autres personnes sont déjà assises

	Aucune (0)	Légère (1)	Moyenne (2)	Sévère (3)
Anxiété	◯	◯	◯	◯

29.

	Jamais: 0% (0)	Occasionnel: 1-33% (1)	Fréquent: 33-67% (2)	Habituel: 67-100% (3)
Evitement	◯	◯	◯	◯

30. Etre le centre d'attention

	Aucune (0)	Légère (1)	Moyenne (2)	Sévère (3)
Anxiété	◯	◯	◯	◯

31.

	Jamais: 0% (0)	Occasionnel: 1-33% (1)	Fréquent: 33-67% (2)	Habituel: 67-100% (3)
Evitement	◯	◯	◯	◯

32. Prendre la parole en réunion

	Aucune (0)	Légère (1)	Moyenne (2)	Sévère (3)
Anxiété	◯	◯	◯	◯

33.

	Jamais: 0% (0)	Occasionnel: 1-33% (1)	Fréquent: 33-67% (2)	Habituel: 67-100% (3)
Evitement	◯	◯	◯	◯

34. Passer un test

	Aucune (0)	Légère (1)	Moyenne (2)	Sévère (3)
Anxiété	◯	◯	◯	◯

35.

	Jamais: 0% (0)	Occasionnel: 1-33% (1)	Fréquent: 33-67% (2)	Habituel: 67-100% (3)
Evitement	◯	◯	◯	◯

36. Exprimer sa divergence ou son désaccord à une personne que vous ne connaissez pas très bien

	Aucune (0)	Légère (1)	Moyenne (2)	Sévère (3)
Anxiété	◯	◯	◯	◯

37.

	Jamais: 0% (0)	Occasionnel: 1-33% (1)	Fréquent: 33-67% (2)	Habituel: 67-100% (3)
Evitement	◯	◯	◯	◯

38. Regarder dans les yeux une personne que vous ne connaissez pas très bien

	Aucune (0)	Légère (1)	Moyenne (2)	Sévère (3)
Anxiété	◯	◯	◯	◯

39.

	Jamais: 0% (0)	Occasionnel: 1-33% (1)	Fréquent: 33-67% (2)	Habituel: 67-100% (3)
Evitement	◯	◯	◯	◯

40. Effectuer un compte-rendu à un groupe

	Aucune (0)	Légère (1)	Moyenne (2)	Sévère (3)
Anxiété	◯	◯	◯	◯

41.

	Jamais: 0% (0)	Occasionnel: 1-33% (1)	Fréquent: 33-67% (2)	Habituel: 67-100% (3)
Evitement	◯	◯	◯	◯

42. Essayer de séduire une personne

	Aucune (0)	Légère (1)	Moyenne (2)	Sévère (3)
Anxiété	◯	◯	◯	◯

43.

	Jamais: 0% (0)	Occasionnel: 1-33% (1)	Fréquent: 33-67% (2)	Habituel: 67-100% (3)
Evitement	◯	◯	◯	◯

44. Rapporter un article dans un magasin

	Aucune (0)	Légère (1)	Moyenne (2)	Sévère (3)
Anxiété	◯	◯	◯	◯

45.

	Jamais: 0% (0)	Occasionnel: 1-33% (1)	Fréquent: 33-67% (2)	Habituel: 67-100% (3)
Evitement	◯	◯	◯	◯

46. Donner une réception

	Aucune (0)	Légère (1)	Moyenne (2)	Sévère (3)
Anxiété	◯	◯	◯	◯

47.

	Jamais: 0% (0)	Occasionnel: 1-33% (1)	Fréquent: 33-67% (2)	Habituel: 67-100% (3)
Evitement	◯	◯	◯	◯

48. Résister à un vendeur très insistant

	Aucune (0)	Légère (1)	Moyenne (2)	Sévère (3)
Anxiété	◯	◯	◯	◯

49.

	Jamais: 0% (0)	Occasionnel: 1-33% (1)	Fréquent: 33-67% (2)	Habituel: 67-100% (3)
Evitement	◯	◯	◯	◯

Partie II : Questionnaire d’acceptation et d’action (AAQ-II)

Vous venez de remplir la partie la plus longue de l'enquête. Veuillez continuer, les parties suivantes sont beaucoup plus courtes !

De plus, à partir de maintenant, vous ne devez plus tenir compte des chiffres entre parenthèses derrière chaque réponse (ceci ne s'appliquait qu'au premier questionnaire).

Voici une liste d’affirmations. Merci d’évaluer à quel point chaque affirmation est vraie pour vous en sélectionnant le chiffre qui correspond le mieux à votre réponse.

1 = Jamais vrai
2 = Très rarement vrai
3 = Rarement vrai
4 = Parfois vrai
5 = Souvent vrai
6 = Presque toujours vrai
7 = Toujours vrai

50.

	Jamais vrai (1)	Très rarement vrai (2)	Rarement vrai (3)	Parfois vrai (4)	Souvent vrai (5)	Presque toujours vrai (6)	Toujours vrai (7)
Mes expériences et mes souvenirs douloureux me gênent pour conduire ma vie comme il me tiendrait à cëur de le faire.	○	○	○	○	○	○	○

51.

	Jamais vrai (1)	Très rarement vrai (2)	Rarement vrai (3)	Parfois vrai (4)	Souvent vrai (5)	Presque toujours vrai (6)	Toujours vrai (7)
J'ai peur de mes émotions.	○	○	○	○	○	○	○

52.

	Jamais vrai (1)	Très rarement vrai (2)	Rarement vrai (3)	Parfois vrai (4)	Souvent vrai (5)	Presque toujours vrai (6)	Toujours vrai (7)
J'ai peur ne pas être capable de contrôler mes inquiétudes et mes émotions.	◯	◯	◯	◯	◯	◯	◯

53.

	Jamais vrai (1)	Très rarement vrai (2)	Rarement vrai (3)	Parfois vrai (4)	Souvent vrai (5)	Presque toujours vrai (6)	Toujours vrai (7)
Mes souvenirs douloureux m'empêchent de m'épanouir dans la vie.	◯	◯	◯	◯	◯	◯	◯

54.

	Jamais vrai (1)	Très rarement vrai (2)	Rarement vrai (3)	Parfois vrai (4)	Souvent vrai (5)	Presque toujours vrai (6)	Toujours vrai (7)
Les émotions sont une source de problèmes dans ma vie.	◯	◯	◯	◯	◯	◯	◯

55.

	Jamais vrai (1)	Très rarement vrai (2)	Rarement vrai (3)	Parfois vrai (4)	Souvent vrai (5)	Presque toujours vrai (6)	Toujours vrai (7)
J'ai l'impression que la plupart des gens gèrent leur vie mieux que moi.	◯	◯	◯	◯	◯	◯	◯

56.

	Jamais vrai (1)	Très rarement vrai (2)	Rarement vrai (3)	Parfois vrai (4)	Souvent vrai (5)	Presque toujours vrai (6)	Toujours vrai (7)
Mes soucis m'empêchent de réussir.	◯	◯	◯	◯	◯	◯	◯

Partie III : Index de sensibilité à l’anxiété – 3 (ASI-3)

Il s'agit du dernier questionnaire. Lorsque vous avez terminé cette partie, il ne reste que quelques courtes questions ouvertes qui vous attendent et vous avez terminé !

Encore une fois, vous n'avez pas besoin de suivre vos scores ici en les écrivant.

S’il vous plaît, sélectionnez la case qui reflète le mieux votre niveau d’accord avec chacun des énoncés suivants.

Si certains des énoncés correspondent à des situations que vous n’avez jamais vécues (exemple : perdre connaissance en public), répondez du mieux que vous pouvez selon ce que vous pensez que vous ressentiriez dans une telle situation. Sinon, répondez à chacun des énoncés selon votre expérience.

Faites attention de ne sélectionner qu’une case pour chaque énoncé et assurez-vous de répondre à chacun des énoncés.

0 = Très peu
1 = Un peu
2 = Moyennement
3 = Beaucoup
4 = Énormément

57.

	Très peu (0)	Un peu (1)	Moyennement (2)	Beaucoup (3)	Énormément (4)
Il est important pour moi de ne pas paraître nerveux(se).					

58.

	Très peu (0)	Un peu (1)	Moyennement (2)	Beaucoup (3)	Énormément (4)
Quand je ne peux pas me concentrer sur une tâche, je m'inquiète de devenir fou / folle.					

59.

	Très peu (0)	Un peu (1)	Moyennement (2)	Beaucoup (3)	Énormément (4)
Cela me fait peur quand mon cœur bat rapidement.					

60.

	Très peu (0)	Un peu (1)	Moyennement (2)	Beaucoup (3)	Énormément (4)
Quand j'ai l'estomac à l'envers, je m'inquiète d'être gravement malade.	◯	◯	◯	◯	◯

61.

	Très peu (0)	Un peu (1)	Moyennement (2)	Beaucoup (3)	Énormément (4)
Cela me fait peur quand je suis incapable de me concentrer sur une tâche.	◯	◯	◯	◯	◯

62.

	Très peu (0)	Un peu (1)	Moyennement (2)	Beaucoup (3)	Énormément (4)
Quand je tremble devant les autres, j'ai peur de ce qu'ils pourraient penser de moi.	◯	◯	◯	◯	◯

63.

	Très peu (0)	Un peu (1)	Moyennement (2)	Beaucoup (3)	Énormément (4)
Quand je ressens un serrement dans ma poitrine, j'ai peur d'être incapable de respirer correctement.	◯	◯	◯	◯	◯

64.

	Très peu (0)	Un peu (1)	Moyennement (2)	Beaucoup (3)	Énormément (4)
Quand je ressens de la douleur à ma poitrine, j’ai peur de faire une crise cardiaque.	○	○	○	○	○

65.

	Très peu (0)	Un peu (1)	Moyennement (2)	Beaucoup (3)	Énormément (4)
Je m'inquiète que les autres remarquent que je suis anxieux(se).	○	○	○	○	○

66.

	Très peu (0)	Un peu (1)	Moyennement (2)	Beaucoup (3)	Énormément (4)
Quand je suis dans la lune, je m'inquiète d’avoir une maladie mentale.	○	○	○	○	○

67.

	Très peu (0)	Un peu (1)	Moyennement (2)	Beaucoup (3)	Énormément (4)
Cela me fait peur quand je rougis devant les autres.	○	○	○	○	○

68.

	Très peu (0)	Un peu (1)	Moyennement (2)	Beaucoup (3)	Énormément (4)
Quand je remarque que mon cœur manque un battement, je m'inquiète qu'il y ait quelque chose qui ne va vraiment pas chez moi.	◯	◯	◯	◯	◯

69.

	Très peu (0)	Un peu (1)	Moyennement (2)	Beaucoup (3)	Énormément (4)
Quand je commence à transpirer dans une situation sociale, j'ai peur que les autres me jugent négativement.	◯	◯	◯	◯	◯

70.

	Très peu (0)	Un peu (1)	Moyennement (2)	Beaucoup (3)	Énormément (4)
Quand mes pensées semblent défiler rapidement, je m'inquiète d'être en train de devenir fou / folle.	◯	◯	◯	◯	◯

71.

	Très peu (0)	Un peu (1)	Moyennement (2)	Beaucoup (3)	Énormément (4)
Quand je sens ma gorge se serrer, j'ai peur de mourir étouffé(e).	◯	◯	◯	◯	◯

72.

	Très peu (0)	Un peu (1)	Moyennement (2)	Beaucoup (3)	Énormément (4)
Quand j'ai de la difficulté à penser clairement, je m'inquiète qu'il y ait quelque chose qui ne va pas chez moi.	◯	◯	◯	◯	◯

73.

	Très peu (0)	Un peu (1)	Moyennement (2)	Beaucoup (3)	Énormément (4)
Je pense qu'il serait horrible de perdre connaissance en public.	◯	◯	◯	◯	◯

74.

	Très peu (0)	Un peu (1)	Moyennement (2)	Beaucoup (3)	Énormément (4)
Quand j'ai un blanc de mémoire, je m'inquiète qu'il y ait quelque chose qui ne va vraiment pas chez moi.	◯	◯	◯	◯	◯

Partie IV : Questions ouvertes

Presque terminé ! Cette section évalue vos expériences avec des pensées, des sentiments et des réactions physiques inconfortables et intrusives.

Il n'y a pas de bonne ou de mauvaise façon de comprendre et de répondre à ces questions. L'idée ici est que vous puissiez élaborer librement sur vos expériences, sans trop vous demander si vous le faites bien ou mal.

Plus vos réponses seront détaillées, plus elles seront précieuses pour notre analyse. Merci de prendre le temps de répondre.

75. Lorsque vous vous trouvez dans une situation sociale que vous percevez comme stressante, ressentez-vous couramment l'une des choses suivantes ?

    -Des pensées indésirables ou inconfortables qui semblent sortir de nulle part.

    -Des sentiments ou des émotions indésirables ou inconfortables.

    -Des réactions corporelles ou des manifestations physiques d'anxiété indésirables ou inconfortables.

    Si oui, veuillez nommer et décrire ces phénomènes.

76. Comment cela vous affecte-t-il lorsque vous remarquez que des pensées, des sentiments ou des réactions corporelles indésirables ou inconfortables surgissent pendant une situation sociale ?

77. Existe-t-il un type spécifique de situation sociale dans laquelle vous ressentez des pensées, des sentiments ou des réactions corporelles indésirables ou inconfortables comme particulièrement perturbantes ?

78. Lorsque vous remarquez que des pensées, des sentiments ou des réactions corporelles indésirables ou inconfortables surgissent au cours d'une situation sociale, comment réagissez-vous habituellement ?

79. Comment la façon dont vous réagissez à des pensées, des sentiments ou des réactions corporelles indésirables ou inconfortables pendant des situations sociales affecte-t-elle votre anxiété ou votre nervosité ?

80. Sélectionnez l'option qui décrit le mieux votre cas.

- Je ressens de la peur ou de l'anxiété dans pratiquement toutes les situations sociales.
- Je ressens de la peur ou de l'anxiété dans un nombre considérable de situations sociales.
- Je ressens de la peur ou de l'anxiété dans une seule ou très peu de situations sociales.
- Aucune de ces réponses

81. Sélectionnez l'option qui décrit le mieux votre cas.

- Dans les situations sociales, je crains surtout de me comporter de manière inepte ou insensée (comme dire quelque chose de déplacé ou quelque chose qui peut être perçu comme "stupide", etc.)
- Dans les situations sociales, je suis surtout préoccupé par le fait de montrer des signes observables d'anxiété (comme une transpiration excessive, le rougissement, une voix tremblante, etc.)
- Dans les situations sociales, je suis principalement préoccupé par la possibilité d'offenser les autres (comme dire quelque chose qui pourrait les blesser, etc.).
- Aucune de ces réponses

82. Veuillez indiquer votre nationalité.

83. Veuillez indiquer votre âge.

This content is neither created nor endorsed by Microsoft. The data you submit will be sent to the form owner.

Microsoft Forms
