# Supplementary material for: Differences in anxiety sensitivity and experiential avoidance between subtypes of social anxiety disorder
Source: PLoS One. 2023 Sep 15;18(9):e0290756. doi: 10.1371/journal.pone.0290756 (PMC10503767; doi:10.1371/journal.pone.0290756)
Supplement: S2 File — Power Analysis, Correlation Matrix, Descriptive Statistics, Regression. (PDF) [file pone.0290756.s002.pdf]

Power Analysis

Experiential Avoidance

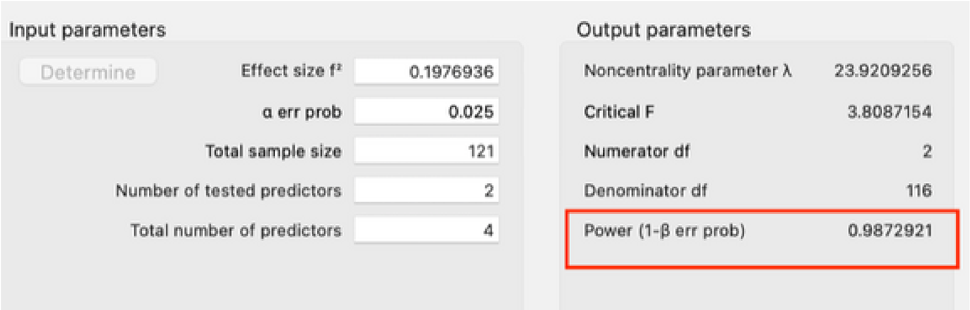

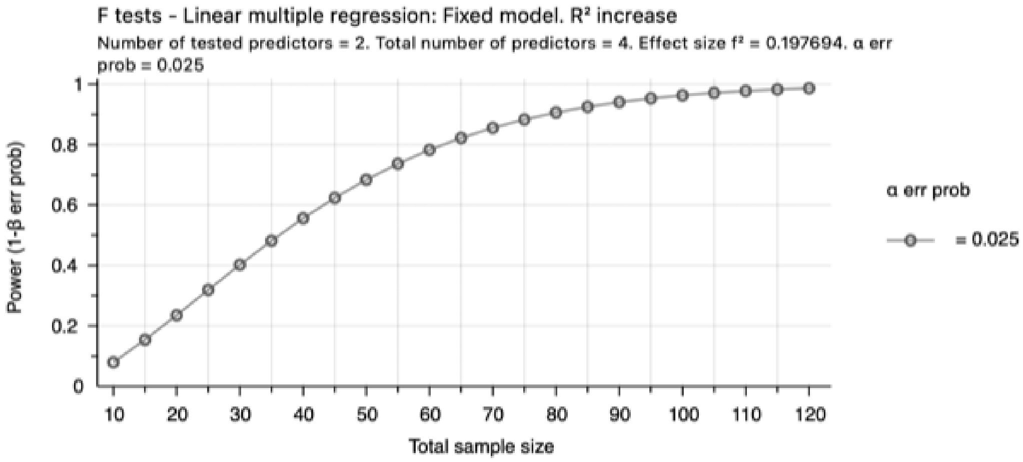

Anxiety Sensitivity

Input parameters		Output parameters	
Effect size f²	0.214168	Noncentrality parameter λ	25.9143280
α err prob	0.025	Critical F	3.8087154
Total sample size	121	Numerator df	2
Number of tested predictors	2	Denominator df	116
Total number of predictors	4	Power (1-β err prob)	0.9924174

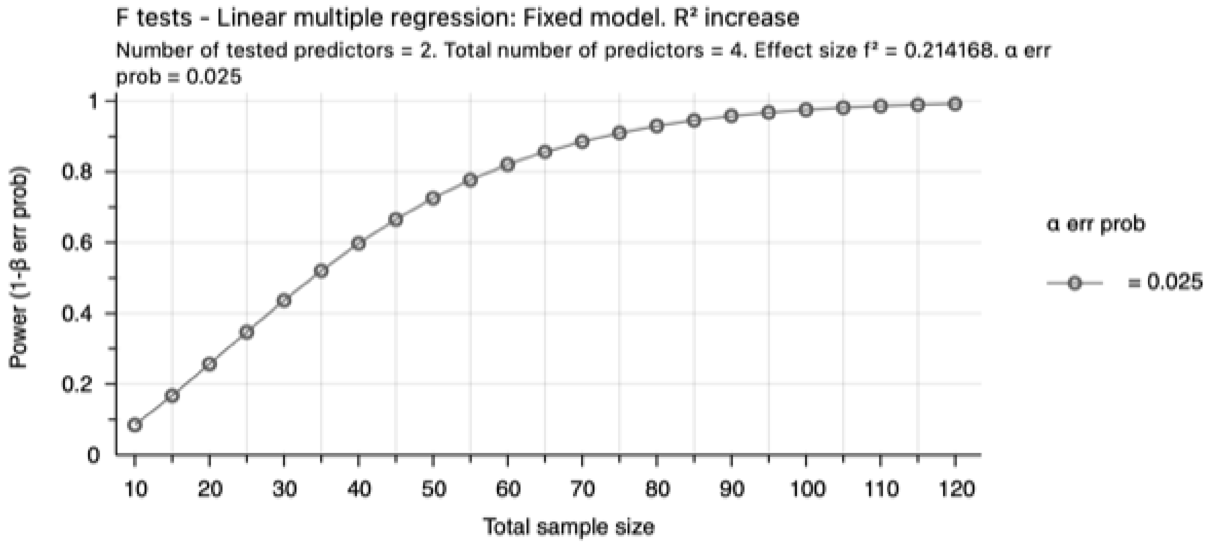

Analysis: Entire Sample

Correlation Matrix

Correlation Matrix

		LSAS SCORE	AAQ Score	ASI Score
LSAS SCORE	Pearson's r	—		
	p-value	—		
AAQ Score	Pearson's r	0.601 ***	—	
	p-value	< .001	—	
ASI Score	Pearson's r	0.509 ***	0.560 ***	—
	p-value	< .001	< .001	—

Note. * p < .05, ** p < .01, *** p < .001

Subscales of Anxiety Sensitivity Index 3

Statistics

		ASI Physical	ASI Social	ASI Cognitive
N	Valid	121	121	121
	Missing	0	0	0
Mean		9.77	17.44	10.26
Std. Deviation		6.382	5.172	6.835
Minimum		0	3	0
Maximum		24	24	24
Percentiles	25	5.00	14.00	4.00
	50	9.00	19.00	10.00
	75	14.00	22.00	16.00

Multiple Linear Regression

Descriptive Statistics

	Mean	Std. Deviation	N
LSAS SCORE	80.91	27.625	116
Age	30.84	11.949	116
HighIncome	.83	.379	116
AAQ Score	33.91	9.450	116
ASI Physical	9.64	6.444	116
ASI Social	17.31	5.214	116
ASI Cognitive	10.27	6.845	116

Variables Entered/Removed[a]

Model	Variables Entered	Variables Removed	Method
1	HighIncome, Age[b]	.	Enter
2	AAQ Score, ASI Social, ASI Physical, ASI Cognitive[b]	.	Enter

a. Dependent Variable: LSAS SCORE

b. All requested variables entered.

Model Summary[c]

Model	R	R Square	Adjusted R Square	Std. Error of the Estimate	Durbin-Watson
1	.061[a]	.004	-.014	27.817	
2	.651[b]	.423	.392	21.547	1.667

a. Predictors: (Constant), HighIncome, Age

b. Predictors: (Constant), HighIncome, Age, AAQ Score, ASI Social, ASI Physical, ASI Cognitive

c. Dependent Variable: LSAS SCORE

ANOVA[a]

Model		Sum of Squares	df	Mean Square	F	Sig.
1	Regression	327.192	2	163.596	.211	.810[b]
	Residual	87436.765	113	773.777		
	Total	87763.957	115			
2	Regression	37158.660	6	6193.110	13.339	.000[c]
	Residual	50605.297	109	464.269		
	Total	87763.957	115			

a. Dependent Variable: LSAS SCORE

b. Predictors: (Constant), HighIncome, Age

c. Predictors: (Constant), HighIncome, Age, AAQ Score, ASI Social, ASI Physical, ASI Cognitive

Coefficients[a]

Model		Unstandardized Coefficients		Standardized Coefficients	t	Sig.	Collinearity Statistics	
		B	Std. Error	Beta			Tolerance	VIF
1	(Constant)	86.324	8.749		9.866	.000		
	Age	-.097	.218	-.042	-.443	.659	.990	1.010
	HighIncome	-2.949	6.873	-.040	-.429	.669	.990	1.010
2	(Constant)	8.176	11.684		.700	.486		
	Age	.030	.171	.013	.178	.859	.969	1.032
	HighIncome	-2.030	5.347	-.028	-.380	.705	.981	1.019
	AAQ Score	1.353	.267	.463	5.067	.000	.634	1.578
	ASI Physical	.245	.479	.057	.512	.609	.425	2.355
	ASI Social	1.542	.492	.291	3.134	.002	.613	1.630
	ASI Cognitive	-.144	.481	-.036	-.300	.765	.372	2.686

a. Dependent Variable: LSAS SCORE

Analysis: Noticeable Symptoms Subtype

Correlation Matrix

Correlation Matrix

		LSAS SCORE	AAQ Score	ASI Score
LSAS SCORE	Pearson's r	—		
	p-value	—		
AAQ Score	Pearson's r	0.710 ***	—	
	p-value	< .001	—	
ASI Score	Pearson's r	0.551 ***	0.622 ***	—
	p-value	< .001	< .001	—

Note. * p < .05, ** p < .01, *** p < .001

Descriptive Statistics for Entire Subtype

Statistics[a]

		LSAS SCORE	AAQ Score	ASI Score	Focus of Fear	Age	HighIncome	ASI Physical	ASI Social	ASI Cognitive
N	Valid	45	45	45	45	45	42	45	45	45
	Missing	0	0	0	0	0	3	0	0	0
Mean		69.82	30.87	34.51	2.00	29.82	.90	9.07	17.56	7.89
Std. Deviation		31.760	10.976	14.106	.000	12.472	.297	5.782	4.240	6.749
Minimum		20	12	11	2	16	0	0	9	0
Maximum		131	49	70	2	58	1	24	24	24
Percentiles	25	45.00	24.00	23.50	2.00	18.50	1.00	5.00	14.50	2.00
	50	68.00	31.00	32.00	2.00	27.00	1.00	8.00	18.00	7.00
	75	97.00	40.50	47.00	2.00	38.00	1.00	13.00	21.00	14.00

a. Focus of Fear = 2

Multiple Linear Regression

Descriptive Statistics[a]

	Mean	Std. Deviation	N
LSAS SCORE	69.98	31.178	42
Age	30.24	12.777	42
HighIncome	.90	.297	42
AAQ Score	30.79	10.694	42
ASI Physical	8.98	5.941	42
ASI Social	17.48	4.346	42
ASI Cognitive	7.86	6.683	42

a. Focus of Fear = 2

Variables Entered/Removed[a,b]

Model	Variables Entered	Variables Removed	Method
1	HighIncome, Age[c]	.	Enter
2	ASI Cognitive, ASI Social, AAQ Score, ASI Physical[c]	.	Enter

a. Focus of Fear = 2

b. Dependent Variable: LSAS SCORE

c. All requested variables entered.

Model Summary[a,d]

Model	R	R Square	Adjusted R Square	Std. Error of the Estimate	Change Statistics					Durbin-Watson
					R Square Change	F Change	df1	df2	Sig. F Change	
1	.067[b]	.004	-.047	31.896	.004	.087	2	39	.917	
2	.721[c]	.520	.438	23.368	.516	9.416	4	35	.000	1.803

a. Focus of Fear = 2

b. Predictors: (Constant), HighIncome, Age

c. Predictors: (Constant), HighIncome, Age, ASI Cognitive, ASI Social, AAQ Score, ASI Physical

d. Dependent Variable: LSAS SCORE

ANOVA[a,b]

Model		Sum of Squares	df	Mean Square	F	Sig.
1	Regression	177.586	2	88.793	.087	.917[c]
	Residual	39677.390	39	1017.369		
	Total	39854.976	41			
2	Regression	20743.427	6	3457.238	6.331	.000[d]
	Residual	19111.550	35	546.044		
	Total	39854.976	41			

a. Focus of Fear = 2

b. Dependent Variable: LSAS SCORE

c. Predictors: (Constant), HighIncome, Age

d. Predictors: (Constant), HighIncome, Age, ASI Cognitive, ASI Social, AAQ Score, ASI Physical

Coefficients[a,b]

Model		Unstandardized Coefficients B	Unstandardized Coefficients Std. Error	Standardized Coefficients Beta	t	Sig.	Correlations Zero-order	Correlations Partial	Correlations Part	Collinearity Statistics Tolerance	Collinearity Statistics VIF
1	(Constant)	75.591	19.443		3.888	.000					
	Age	.023	.390	.009	.059	.953	.007	.009	.009	.998	1.002
	HighIncome	-6.979	16.783	-.067	-.416	.680	-.066	-.066	-.066	.998	1.002
2	(Constant)	-3.440	22.937		-.150	.882					
	Age	-.057	.299	-.023	-.191	.849	.007	-.032	-.022	.913	1.096
	HighIncome	-3.414	12.470	-.033	-.274	.786	-.066	-.046	-.032	.970	1.031
	AAQ Score	1.605	.466	.550	3.445	.002	.685	.503	.403	.537	1.864
	ASI Physical	.644	.935	.123	.688	.496	.463	.116	.081	.431	2.319
	ASI Social	1.362	1.011	.190	1.347	.187	.500	.222	.158	.690	1.449
	ASI Cognitive	-.095	.926	-.020	-.102	.919	.513	-.017	-.012	.347	2.879

a. Focus of Fear = 2

b. Dependent Variable: LSAS SCORE

Analysis: Inept Behavior Subtype

Correlation Matrix

Correlation Matrix

		LSAS SCORE	AAQ Score	ASI Score
LSAS SCORE	Pearson's r	—		
	p-value	—		
AAQ Score	Pearson's r	0.424 ***	—	
	p-value	< .001	—	
ASI Score	Pearson's r	0.439 ***	0.490 ***	—
	p-value	< .001	< .001	—

Note. * p < .05, ** p < .01, *** p < .001

Descriptive Statistics

Statistics[a]

		LSAS SCORE	AAQ Score	ASI Score	Focus of Fear	Age	HighIncome	ASI Physical	ASI Social	ASI Cognitive
N	Valid	66	66	66	66	66	64	66	66	66
	Missing	0	0	0	0	0	2	0	0	0
Mean		89.33	36.35	40.62	3.00	30.89	.77	10.53	18.20	11.89
Std. Deviation		23.662	8.062	16.673	.000	10.822	.427	6.828	5.438	6.582
Minimum		39	11	3	3	17	0	0	3	0
Maximum		130	49	72	3	62	1	24	24	24
Percentiles	25	70.00	32.75	30.75	3.00	22.75	1.00	5.00	14.75	7.00
	50	92.50	38.00	41.00	3.00	28.00	1.00	10.00	20.00	11.50
	75	111.00	42.00	51.50	3.00	37.50	1.00	15.25	23.00	16.00

a. Focus of Fear = 3

Multiple Linear Regression

Descriptive Statistics[a]

	Mean	Std. Deviation	N
LSAS SCORE	88.70	23.742	64
Age	30.67	10.585	64
HighIncome	.77	.427	64
AAQ Score	36.38	8.011	64
ASI Physical	10.34	6.850	64
ASI Social	18.05	5.452	64
ASI Cognitive	11.86	6.662	64

a. Focus of Fear = 3

Variables Entered/Removed[a,b]

Model	Variables Entered	Variables Removed	Method
1	HighIncome, Age[c]	.	Enter
2	ASI Physical, AAQ Score, ASI Social, ASI Cognitive[c]	.	Enter

a. Focus of Fear = 3

b. Dependent Variable: LSAS SCORE

c. All requested variables entered.

Model Summary[a,d]

Model	R	R Square	Adjusted R Square	Std. Error of the Estimate	Change Statistics					Durbin-Watson
					R Square Change	F Change	df1	df2	Sig. F Change	
1	.036[b]	.001	-.031	24.113	.001	.040	2	61	.961	
2	.635[c]	.403	.341	19.280	.402	9.602	4	57	.000	1.508

a. Focus of Fear = 3

b. Predictors: (Constant), HighIncome, Age

c. Predictors: (Constant), HighIncome, Age, ASI Physical, AAQ Score, ASI Social, ASI Cognitive

d. Dependent Variable: LSAS SCORE

ANOVA[a,b]

Model		Sum of Squares	df	Mean Square	F	Sig.
1	Regression	46.464	2	23.232	.040	.961[c]
	Residual	35466.895	61	581.425		
	Total	35513.359	63			
2	Regression	14324.560	6	2387.427	6.422	.000[d]
	Residual	21188.799	57	371.733		
	Total	35513.359	63			

a. Focus of Fear = 3

b. Dependent Variable: LSAS SCORE

c. Predictors: (Constant), HighIncome, Age

d. Predictors: (Constant), HighIncome, Age, ASI Physical, AAQ Score, ASI Social, ASI Cognitive

Coefficients[a,b]

Model		Unstandardized Coefficients		Standardized Coefficients	t	Sig.	Correlations			Collinearity Statistics	
		B	Std. Error	Beta			Zero-order	Partial	Part	Tolerance	VIF
1	(Constant)	89.913	10.206		8.810	.000					
	Age	-.071	.290	-.032	-.245	.807	-.028	-.031	-.031	.978	1.022
	HighIncome	1.266	7.194	.023	.176	.861	.018	.023	.023	.978	1.022
2	(Constant)	12.987	15.917		.816	.418					
	Age	.109	.236	.049	.464	.645	-.028	.061	.047	.949	1.053
	HighIncome	-1.712	5.806	-.031	-.295	.769	.018	-.039	-.030	.960	1.041
	AAQ Score	1.050	.353	.354	2.973	.004	.435	.366	.304	.737	1.357
	ASI Physical	.013	.554	.004	.024	.981	.341	.003	.002	.409	2.444
	ASI Social	2.622	.626	.602	4.186	.000	.546	.485	.428	.506	1.977
	ASI Cognitive	-1.010	.583	-.283	-1.733	.088	.279	-.224	-.177	.392	2.554

a. Focus of Fear = 3

b. Dependent Variable: LSAS SCORE
